# Purinergic ecto-enzyme CD73 is a context-dependent tumor suppressor in colorectal cancer

**DOI:** 10.1016/j.jbc.2025.110864

**Published:** 2025-10-27

**Authors:** Tieu Lan Chau, Ümran Borucu, Beste Uygur, Ahmet Göktuğ Özkurt, Eylül Kılıç, Aynur Işık, Ayşe Dila Gemalmayan, Caglar Cekic, Aytekin Akyol, Seçil Demirkol Canlı, Serkan İsmail Göktuna

**Affiliations:** 1 Department of Molecular Biology and Genetics, Bilkent University, Ankara, Türkiye; 2 Transgenic Animal Technologies Research and Application Center, Hacettepe University, Ankara, Türkiye; 3 Department of Pathology, Hacettepe University Faculty of Medicine, Ankara, Türkiye; 4 Molecular Pathology Application and Research Center, Hacettepe University, Ankara, Türkiye; 5 Tumor Pathology, Cancer Institute, Hacettepe University, Ankara, Türkiye

**Keywords:** cell death, colorectal cancer, purinergic signaling, CD73/NT5E, tumor suppressor.

## Abstract

Inhibition of purinergic signaling in cancer has recently received great attention. The purinergic ecto-enzyme CD73 represents a prominent candidate; however its intrinsic role in colorectal cancer (CRC) cells has not been fully investigated. Stable depletion of CD73 expression by using CRISPR/Cas9 in CRC cell lines led to increased cell proliferation, enhanced cell motility and the formation of larger xenograft tumors in mice. These observations may be explained by an exacerbation of the EMT process. In addition, acquired resistance to gefitinib, an EGFR inhibitor, in various CRC models is associated with the loss of CD73 expression. Overexpression of CD73 in CRC cells can revert some of these phenotypes, resulting in slower cell migration, forming smaller xenograft tumors, sensitizing gefitinib response and enhanced apoptosis. Further supporting the tumor suppressive roles of CD73, its overexpression in CRC cells increased vulnerability to cell death induced by multiple agents while its depletion provided protection. Moreover, our bioinformatic analyses with human patient samples supported our *in vitro* and *in vivo* results, indicating that the tumor suppressor function of CD73 depends on stromal content and infiltrating immune cell. Collectively, our data strongly reveal that CD73 can function as a tumor suppressor in CRC cells. Therefore, inhibition of CD73 in general may not bring the expected outcome in patient subgroups with poor immune cell infiltration highlighting the need for careful evaluation and personalized treatment based on histopathological features of tumors.

Colorectal tumorigenesis is a complex process, involving both genetic and environmental elements. Furthermore, colorectal cancer (CRC) can represent an excellent example of tumor evolution in which the tumor microenvironment (TME), together with mutated cells, can play an important role in the initiation, promotion and progression of cancer (1). Since CRC has long been associated with chronic inflammation (2), modulation of inflammatory immune responses has emerged as a new treatment strategy for CRC in recent years (3).

Adenosine, an ATP-derived molecule, is one of the most critical regulatory factors in the TME (4). Cellular activities or stress signals such as injury, hypoxia or cell death cause ATP to release to extracellular space, inducing inflammatory responses through the activation of P2 purinergic receptors found on various immunocytes (5). Since exacerbated immune responses may lead to excessive cell death and tissue damage, ATP should be quickly converted to adenosine which then acts as an anti-inflammatory agent through the binding to P1 purinergic receptors to induce anti-inflammatory signaling pathways. However, within tumor microenvironment, extracellular adenosine signaling can be exploited by tumor cells to suppress immunocyte activity, a process known as “immune escape”, which allows tumors to evade immunosurveillance (6, 7). To be converted to adenosine, ATP is first catalyzed to ADP or AMP by the extracellular ectonucleotidases (CD39 and CD73 respectively) found on the surface of various cells including T cells, endothelial cells and myeloid cells (8, 9). CD73, a 70 kDa cell surface protein, has also been found overexpressed in many types of cancer and shown to be a key regulatory molecule for cell proliferation, migration and invasion, tumor angiogenesis, and tumor immunotolerance *in vivo* (10).

Although CD73 has become an appealing therapy target in various pathologies (11, 12, 13) an association of high CD73 expression with good prognosis has been reported in some cancers (14, 15). The correlation of CD73 expression with clinical outcome may depend on the cell types in TME that express CD73 (16, 17). Indeed, our bioinformatic analysis have also revealed some contradictory findings on roles of CD73 in CRC Given that mechanistic studies of CD73 in CRC are limited, our aim was to elucidate the intrinsic roles of CD73 in cancer. To achieve this, we examined the effects of CD73 loss or gain in CRC cell lines using both *in vitro* and *in vivo* models. Specifically, we generated CD73 loss-of-function CRC cell line models with the CRISPR/Cas9 gene editing system, and employed transient and stable CD73 overexpression as gain-of-function models. Experiments with at least two different CD73-deficient CRC cell lines showed the same effects in promoting cell growth, cell motility and inducing EMT, whereas its overexpression reverted these phenotypes. Our study revealed that CD73 rather acts as a tumor suppressor in colorectal tumor cells. This finding was reinforced when CD73 protein expression was significantly reduced in gefitinib, an EGFR inhibitor, and resistant CRC cell lines. Finally, our deep bioinformatic analyses with patient data have shed more light on the tumor suppressive role of CD73 in CRC. High CD73 expression is associated with worse prognosis only in patients whose tumors have high stromal involvement and immune infiltrates. These effects are reversed in patients with tumors showing low levels of stroma and immune cell association.

## Results

Since CD73 is encoded by the NT5E gene, throughout the manuscript, “NT5E” will be used whenever we mentioned about the gene, gene constructs, RNA interference or mRNA expressions. On the other hand, “CD73” will be used for the protein or protein expression from NT5E gene.

### Bioinformatic analysis on CD73 expression in CRC patients reveal contradicting roles of CD73 in colorectal cancer

**Figure 1:**
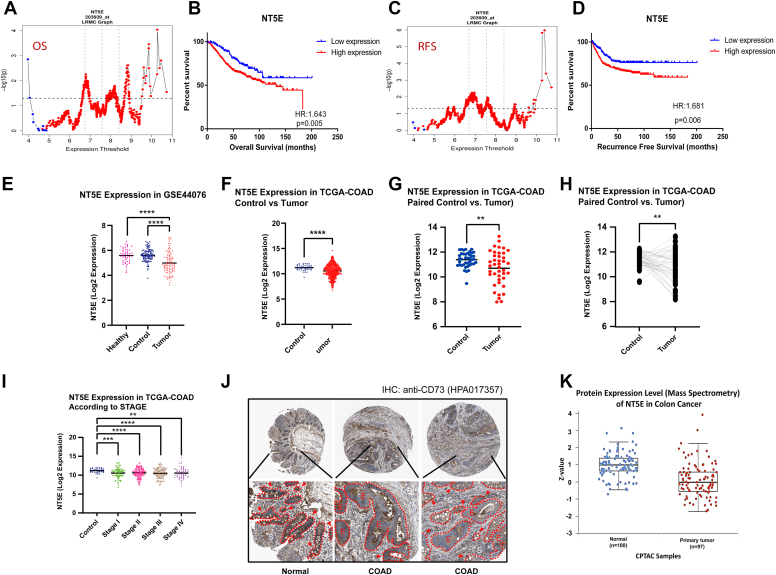
**Bioinformatic analysis on CD73 expression in CRC patients reveal contradicting role of CD73 in colorectal cancer.** 203939_at probeset is used for *NT5E* expression in the GSE39582 dataset. *A* and *B*, high *NT5E* expression is associated with poor overall survival according to Log-rank multiple cut-off^#^(LRMC) graph (*A*) and Kaplan-Meier (KM) plot (*B*). The expression threshold at which the lowest log-rank *p* value is obtained (6.786532) within interquartile range was utilized to generate high (n = 372) and low (n = 147) expression groups. *C* and *D*, high *NT5E* expression is associated with poor and recurrence-free survival (RFS) according to according to LRMC graph (*C*) and KM plot (*D*). The expression threshold at which the lowest log-rank *p* value is obtained (6.961637) within interquartile range was utilized to generate high (n = 342) and low (n = 177) expression groups. *E*, *NT5E* expression (average of 11719174_a_at, 11744681_a_at, 11755207_a_at probesets) in healthy individual *versus* paired normal and tumor samples of colorectal cancer (CRC) patients in GSE44076 microarray dataset. Healthy (n = 50), Control (n = 98) and Tumor (n = 98) (∗∗∗∗*p* < 0.0001). *F*, NT5E expression in primary tumor and normal colon samples in TCGA COAD patient samples. Tumor (n = 456), Control (n = 41) (∗∗∗∗*p* < 0.0001) (*G* and *H*) *NT5E* expression in paired normal *versus* tumor samples of TCGA COAD patients in paired dot plot with different presentation formats. *I*, *NT5E* expression in patient samples according to the disease stage from TCGA-COAD dataset; Tumor (n = 456), Control (n = 41). *J*, CD73 protein expression was measured *via* immunohistochemistry (IHC) using antibodies against CD73 (HPA017357) in normal *versus* tumor (CRC) samples from The Human Protein Atlas. Patient IDs: healthy (3266), CRC-1 (2001), and CRC-2 (2096). *Red dashed lines* and red arrows indicate epithelial cells (*E*) while *white asterixis* show stromal (S) areas with high CD73 expression. *K*, CD73 protein expression in Clinical Proteomic Tumor Analysis Consortium patient samples as measured by mass spectrometry analysis (*p* = 3.72654150065602E-11); normal (n = 100), primary tumor (n = 97). Proteomics results presented here obtained from UALCAN platform. ^#^ LRMC graph shows gene expression-based cut-offs on the x axis and log-rank *p* values at each specific cut-off on the y axis. The threshold within the interquartile range that gives the lowest log-rank *p* value (6.96) was used to categorize low and high expression groups. For all LRMC graphs: *blue* and *red colors* indicate association with good and poor prognosis, respectively; *vertical dashed lines* represent 25th percentile, median and 75th percentile values. *Horizontal dashed line* indicates 0.05 *p* value. IHC, immunohistochemistry; RFS, recurrence-free survival; LRMC, log-rank multiple cut-off; KM, Kaplan-Meier; CRC, colorectal cancer.

Elevated levels of CD73 were reported in many cancers such as breast (13, 18), prostate (19, 20), and lung cancer (21). However, its comprehensive characterization in CRC was not adequate. For this reason, we wanted to gather further evidence through both bioinformatic analysis and experiments. In a large cohort of CRC patients (GSE39582), *NT5E* high expression was significantly associated with poor overall (OS) and recurrence-free survival (RFS) (Fig. 1, *A* and *C*). It was consistent at multiple cut-offs that determine groups with high and low expression as also evident in Kaplan Meier survival graphs (Fig. 1, *B* and *D*). These data suggested that CD73 functions as an oncogene.

However, using transcriptomic data available from independent public datasets, we observed that *NT5E* expression was significantly lower in colon tumors compared to both adjacent normal mucosa and healthy colon (Fig. 1*E*) when expression of *NT5E* was compared between colon mucosa obtained from healthy individuals, colon tumors and adjacent paired normal mucosa from CRC patients. Similarly, with the RNA-Seq data available from TCGA-COAD samples, CRC tumor samples showed lower *NT5E* expression than adjacent normal tissues when analyzed in both unpaired (Fig. 1*F*) and paired (Fig. 1, *G* and *H*) fashions. When we checked *NT5E* expression in tumors with different disease stage, all stages were showing lower *NT5E* expression than control tissues (Fig. 1*I*). Supporting these transcriptomic data, staining from pathological specimens of healthy colon tissues https://www.proteinatlas.org/ENSG00000135318-NT5E/tissue/colon#img showed higher CD73 levels in epithelial cell compartment than tumor samples in colon cancer (COAD) patients https://www.proteinatlas.org/ENSG00000135318-NT5E/cancer/colorectal+cancer#img from the Human Protein Atlas (Fig. 1*J*). However, the stromal compartment in the tumor samples exhibited higher CD73 stainings compared to the stroma of normal tissues. In addition, we analyzed CD73 protein expression in normal *versus* CRC specimens using data from Clinical Proteomic Tumor Analysis Consortium *via* the UALCAN platform (22). Analysis of mass spectrometry-based protein expression data further supported the reduction of CD73 expression in colorectal tumor samples (Fig. 1*K*). Collectively, these results strongly suggest that CD73 expression is reduced in colorectal tumors compared to healthy tissues, primarily due to decreased protein levels in epithelial cells.

### CD73 is abundantly expressed in cancer cells in tumors and in most CRC cell lines

**Figure 2:**
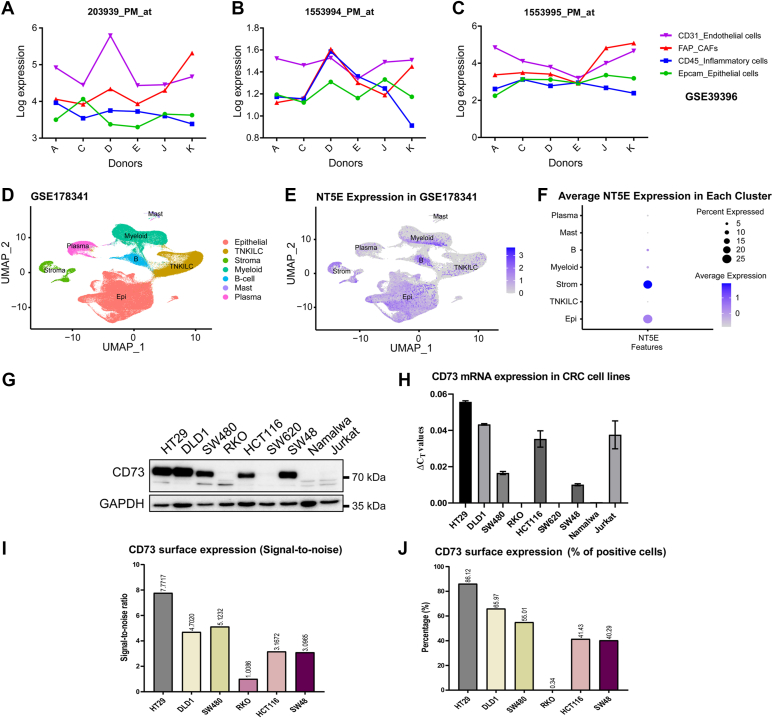
**CD73 is abundantly expressed in cancer cells in tumors and in most CRC cell lines.***A* and *C*, expression of CD73 in FACS sorted cells obtained from 6 CRC tumors (donors *A*–*K*). Results from 3 different probesets of CD73 (203939_PM_at, 1553994_PM_at and 1553995_PM_at). ∗Log2 intensities are obtained from normalized microarray data; therefore, the values are absolute not relative. *D* and *F*, UMAP based visualization of GSE178318 dataset which contain 6 primary tumor samples of CRC patients. Clusters were either colored according to (*D*) different cell populations or (*E*) *NT5E* expression levels (*F*) Average *NT5E* expression in each cluster in UMAP was also visualized *via dot plo*ts. *G*, CD73 protein expression levels from seven different CRC cell lines compared to that of two immortalized immune cell lines such as Namalwa (B lymphocytes) and Jurkat (T lymphocytes) *via* Western blot. *H*, *NT5E* mRNA levels were measured by qPCR in the same cell lines that were checked with Western blotting. *(I* and *J*,) cell surface expression profile of CD73 on indicated CRC cells analyzed by flow cytometry, reflecting (*I*) the intensity of CD73 signal (signal-to-noise ratio) and (*J*) its density in the cell population (% of the cells expressing CD73), which is in line with the Western blot result. CRC, colorectal cancer.

Next, we wanted to identify the cell types in CRC tumors that express *NT5E*. Three probesets annotated for NT5E were analyzed in FACS sorted cells from 6 CRC donors in the GSE39396 dataset (Fig. 2, *A*–*C*). Although *NT5E* levels from the sorted donor samples did not show a consistent pattern in different cell types, the two probesets with the highest mean expression (203939_PM_at, 1553995_PM_at) showed that *NT5E* expression was high in CAFs and endothelial cells in most donors. On the other hand, *NT5E* expression in epithelial cells were also about the same level in most donor samples (according to two probes with highest mean).

In another analysis with scRNA-Seq data from 63 CRC patient tumor samples in the GSE178341 dataset (Fig. 2, *D*–*F*), standard Seurat workflow (23) was followed and annotated cell types were visualized by UMAP (Fig. 2*D*). The *NT5E* expression in each cell cluster was shown *via* UMAP (Fig. 2*E*) or weighed dot plot (Fig. 2*F*). In line with the FACS sorted cell expression data, *NT5E* expression in scRNA-Seq cluster showed that stromal cells had the highest *NT5E* expression followed by epithelial cells. Similar analysis was done in another dataset (GSE178318) with 6 tumor samples (Fig. S1, *A*–*C*). Again, epithelial and stromal cells were showing the most abundant *NT5E* expression, albeit epithelial cells had the most *NT5E* expression in these samples. Therefore, single-cell expression data from two independent datasets consistently showed that *NT5E* is abundantly expressed in cancer cells in CRC tumors.

Finally, we wanted to check CD73 protein (Fig. 2*G*) and mRNA (Fig. 2*H*) expressions in seven different CRC cell lines. Immortalized T lymphocyte cell line Jurkat and immortalized B lymphocyte cell line Namalwa was included as references since CD73 was reportedly well expressed (24).

Western blot results showed that CD73 was highly expressed in most CRC cell lines, except RKO and SW620, while it was barely detected in the immortalized lymphocyte cell lines as reported previously (25). This could be because the immortalized cell lines were not activated to be fully functional as the mRNA level in Jurkat was indeed very high. Their CD73 expression, therefore, can be considered as background levels. Among these CRC cell lines, CD73 expressions were higher in HT29 and DLD1 than in the rest of the cell lines. WB analysis of CD73 expression in various CRC cell lines was repeated three times by including non-malignant colon epithelial cell (CCD-18Co) extracts (Fig. S1*D*). The results were also presented as normalized expression graphs (Fig. S1*E*).

Since CD73 is a cell surface protein, we wanted to confirm its expression profile at the cell surface *via* flow cytometry. The intensity of CD73 in each cell line is reflected by signal to noise ratios which are calculated by dividing Mean Fluorescence Intensityvalues of positive (stained) samples by that of negative (unstained) samples (Fig. 2*I*). The percentage of CD73 expressing cells in a given population was also presented (Fig. 2*J*). Indeed, CD73/NT5E was highly expressed on the surface of HT-29 and DLD-1 cells in line with the results of Western blot analyses. Detailed flow cytometry histograms and dot-plots related to CD73 surface expression in CRC cells are shown (Fig. S1, *F*–*I*).

### CD73-deficient CRC cells exhibit enhanced proliferation *in vitro* and *in vivo*

**Figure 3:**
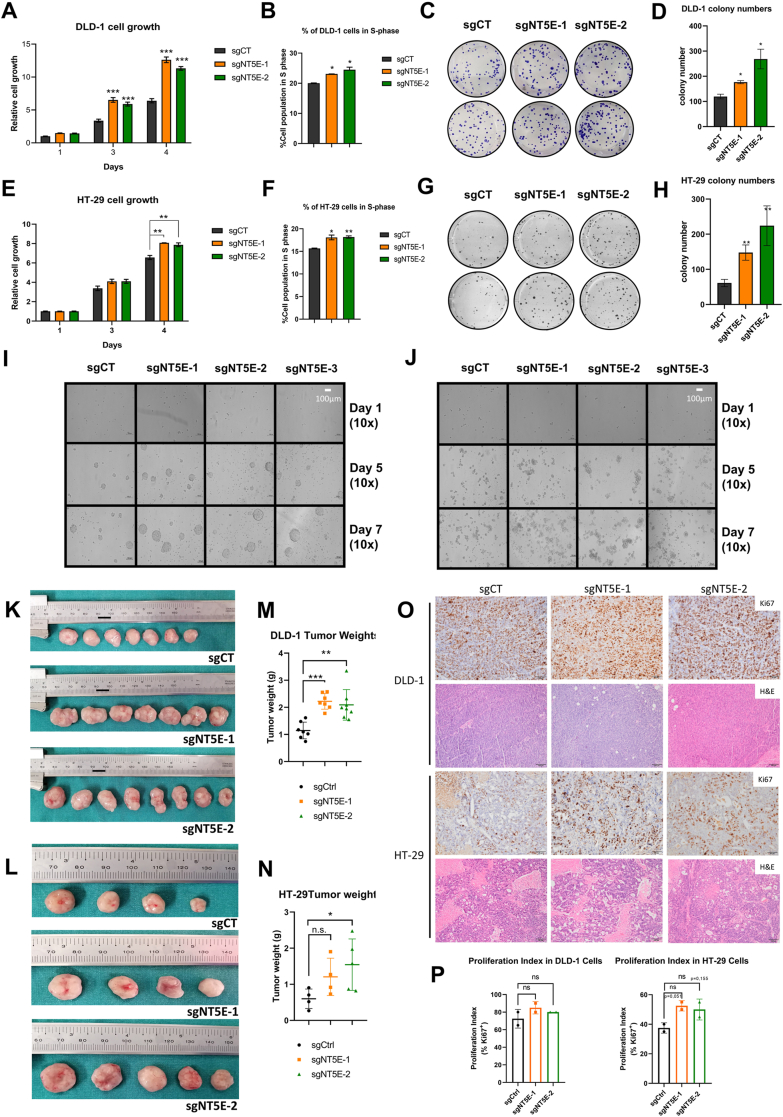
**CD73 expression loss enhances CRC cell proliferation, clonogenicity and anchorage-independent growth and lead to larger xenografts than the ones of their controls.***A* and *B*, cell proliferation assay of control or CD73-deficient (*A*) DLD-1 and (*B*) HT-29 cells. Cells were seeded into 96 well plates, triplicate for each condition, then viable cells were quantified every day thereafter by using Cell Tilter Glo kit which measured ATP amount of metabolically active cells. Relative cell growth of each condition was calculated by dividing their values to the ones of the control cell of the first day. *C* and *D*, percentage of cell population in S phase of control or CD73-deficient (C) DLD-1 and (D) HT-29 cells from the flow cytometry analysis were graphed. (∗*p* < 0.05, ∗∗*p* < 0.01) for both cell lines. *E* and *H*, colony formation assay and quantification graphs of control or CD73-deficient (*E*–*G*) DLD-1 and (*F*–*H*) HT-29 cells. *I* and *J*, anchorage-independent colonies of CD73-deficient CRC (*I*) DLD-1 and (*J*) HT-29 cells that were plated onto polyHema-coated 96 well plates and colony formation was pictured over the indicated times. CD73 depleted cells were showing more numerous and bigger colonies as compared to their control cells for both cell lines. *K* and *L*, xenograft tumors of control or CD73-deficient (*K*) DLD-1 (sgNT5E-DLD-1) and (*L*) of HT-29 (sgNT5E-HT-29) cells that were injected subcutaneously to the flanks of athymic nude mice. *M* and *N*, tumor weights at the time of sacrifice also showed significant differences for both constructs in DLD-1 (*p* < 0.001 and 0.0017, respectively) and only in one construct in HT-29 (*p* = 0.0829 and 0.0421, respectively). ∗*p* < 0.05; ∗∗*p* < 0.01; ∗∗∗*p* < 0.001 for both cell lines. *O*, Ki67 and H&E histological stainings in xenograft tumor samples of control and CD73-deficient DLD-1 and HT-29 cells (this scale bar represent100 μm). *P*, proliferation indices based on Ki67 IHC stainings in xenograft tumor samples of control and CD73-deficient DLD-1 and HT-29 cells, represented as bar graphs. IHC, immunohistochemistry; CRC, colorectal cancer.

To investigate the intrinsic roles of CD73 in CRC cell lines, by using CRISPR/Cas9 (sgNT5E) technology, we first generated CD73-deficient CRC cell lines (Fig. S2) then tested the proliferative abilities of control and CD73-deficient cell lines by cell viability assay. Loss of CD73 resulted in increased cell proliferation in DLD-1 (Fig. 3*A*) and HT29 (Fig. 3*B*) cells. To reinforce this result, cell cycle analysis was performed *via* flow cytometry. Indeed, the S phase population of sgNT5E-DLD-1 (Figs. 3*C* and S2*I*) and of sgNT5E-HT-29 (Figs. 3*D* and S2*J*) cells were significantly higher than their control cells. Next, clonogenic assays were carried out to assess the ability of sgNT5E-depleted cell lines to form colonies from a single cell. We observed that CD73 depletion increases the ability of DLD-1 (Fig. 3, *E* and *F*) and HT-29 (Fig. 3, *G* and *H*) to form more colonies. These findings ultimately support that CD73 depletion in CRC cells increased cell proliferation.

To investigate the anchorage independence of CD73-deficient CRC cells, we coated 96-well plates with a hydrogel called polyHEMA to prevent cellular attachment and allow cells to grow into spheres of colonies. The changes in cell growth and morphology were evident from day 5 or day 7 in CRC cell lines. sgNT5E-DLD-1 cells (Fig. 3*I*) and HT-29 cells (Fig. 3*J*), produced colonies that were larger and more numerous than their controls. In summary, CD73 loss affected CRC cell growth abilities.

Finally, to confirm the enhanced proliferation *in vitro*, CD73-deficient CRC cell lines were injected subcutaneously into athymic nude mice. Tumor volumes were recorded three times a week (Fig. S2, *K* and *L*) and experiments were terminated when tumor sizes were reached to humane conditions (15 mm in diameter or approx. 1500 mm^3^). Tumor growth in CD73-deficient cells was observed to be faster than controls. At the time of the sacrifice CD73-deficient DLD-1 and HT-29 cells developed much larger tumors than their control cells as observed by tumor pictures (Fig. 3, *K* and *L*) and tumor weights (Fig. 3, *M* and *N*). Most of these differences in tumor sizes (with the exception of sgNT5E-1 in HT-29, *p* = 0.0829) were statistically significant. Moreover, we have measured proliferation in these tumors using Ki67 immunohistochemistry (Fig. 3, *O* and *P*). We observed that CD73-deficient xenograft generally exhibited higher level of Ki67 staining compared to their respective controls (Fig. 3*O*). This increase in proliferative index was more pronounced in HT-29 xenografts than in DLD-1 xenografts with CD73 deficiency (Fig. 3*O*). Although these results did not reach statistical significance (likely due to small sample size, n = 2), they still support the notion that CD73 deficiency contributes to increased proliferation of CRC cells both *in vitro* and in *vivo*.

### Enhanced migration and invasion of CRC cell lines upon CD73 loss of expression is linked to enhanced EMT signature

**Figure 4:**
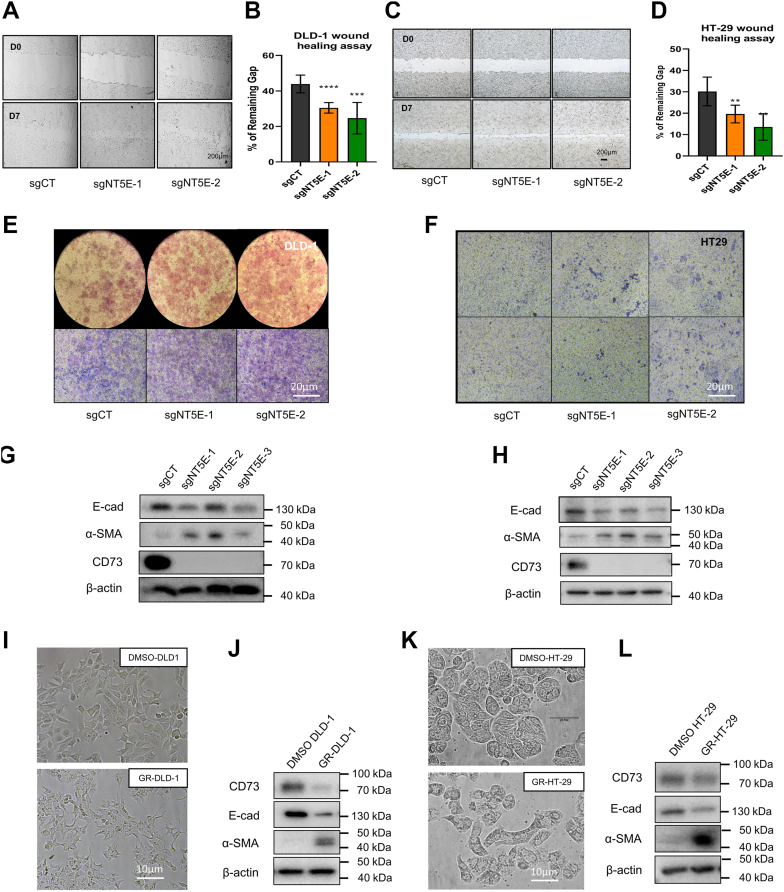
**CD73 loss of expression promoted migration and invasion of CRC cell lines, involving the EMT process.***A* and *D*, wound healing assay and quantitative measurement of the gap closures of control *versus* CD73-deficient (*A* and *B*) DLD-1 after 36 h and (*C* and *D*) HT-29 after 7 days. CD73-deficient cells moved faster than their control group in both cell lines. *E* and *F*, transwell Invasion assay with control *versus* CD73-deficient (*E*) DLD1 or (*F*) HT-29 cells. Transwells were precoated with a thin layer of Matrigel before seeding the cells in 1% Fetal Bovine Serum media. 20% Fetal Bovine Serum media were added to the outer chamber to create cell attraction force. Transwells of DLD1 cells were stained after 24 h, and of HT-29 cells were stained after 48 h. Pictures were taken by camera (*E-up*) and inverted microscope (*E-down* and *F*) at representative fields of the wells. The CD73-deficient cells were also more invasive as compared to their control groups. *G* and *H*, loss of CD73 expression is associated with enhanced EMT signature in DLD1 (*G*) and HT29 (*H*) cells. *I* and *L*, Cell morphology and their EMT profile by Western blots of gefitinib resistant DLD1 (*I*–*J*) and HT29 (*K*–*L*) cell lines.

To test the migratory abilities of CRC cells upon CD73 loss, the wound healing assay was conducted. CD73-depleted cells sgNT5E-DLD-1 (Fig. 4, *A* and *B*) and sgNT5E-HT-29 (Fig. 4, *C* and *D*) could migrate faster than their control ones. To assess the invasive ability of these CD73-deficient cells, we used the transwells precoated with a thin layer of Matrigel. CD73-deficient CRC cells invaded more efficiently than their controls (Fig. 4, *E* and *F*).

To explain the improved proliferative and migration abilities of sgNT5E-CRC cells, we investigated mRNA and protein levels of some of the EMT markers. Indeed, upon CD73 loss, EMT was induced in DLD-1 (Fig. 4*G*), and HT-29 (Fig. 4*H*) cells as evidenced by a reduction of E-cadherin expression and an induction of alpha smooth actin, although not all EMT markers were similarly affected (Fig. S3, *A* and *B*), probably due to cell line specificity. EMT marker expression profiles at mRNA levels were also presented (Fig. S3*C*). sgNT5E-HT-29 cells showed slight decrease in epithelial markers including *CDH1*, *TJP1* (*ZO-1)*, and *CLDN7* (*Claudin-7)* while mesenchymal markers such as vimentin and *ZEB1* were increased. *CDH2*, *ZEB2*, and *TWIST1* mRNA levels were found to be significantly augmented. Interestingly, in sgNT5E-HT29 cells, ERK1/2 and AKT phosphorylation were considerably enhanced whereas total expressions of these proteins were unchanged (Fig. S3*B*).

Given the correlation between CD73 deficiency and enhanced EMT signature, we questioned whether CD73 level would also decline in acquired drug resistance cell models where EMT induction is usually seen as a hallmark. For this purpose, we checked CD73 expression in different gefitinib resistant (GR) CRC cell lines created in the lab (Fig. S3, *D*–*G*). A significant reduction in CD73 level was seen in these (GR) CRC cell lines with enhanced EMT (Fig. S3, *F* and *G*). The changes in EMT markers upon drug resistance (Fig. 4, *J* and *L*) were consistent with the EMT profiles of CD73-deficient cell lines (Fig. 4, *G* and *H*).

### Overexpression of CD73 in CRC cells displays slower cell growth *in vitro* and *in vivo*, induces apoptosis, hampers cell migration and sensitizes gefitinib response

**Figure 5:**
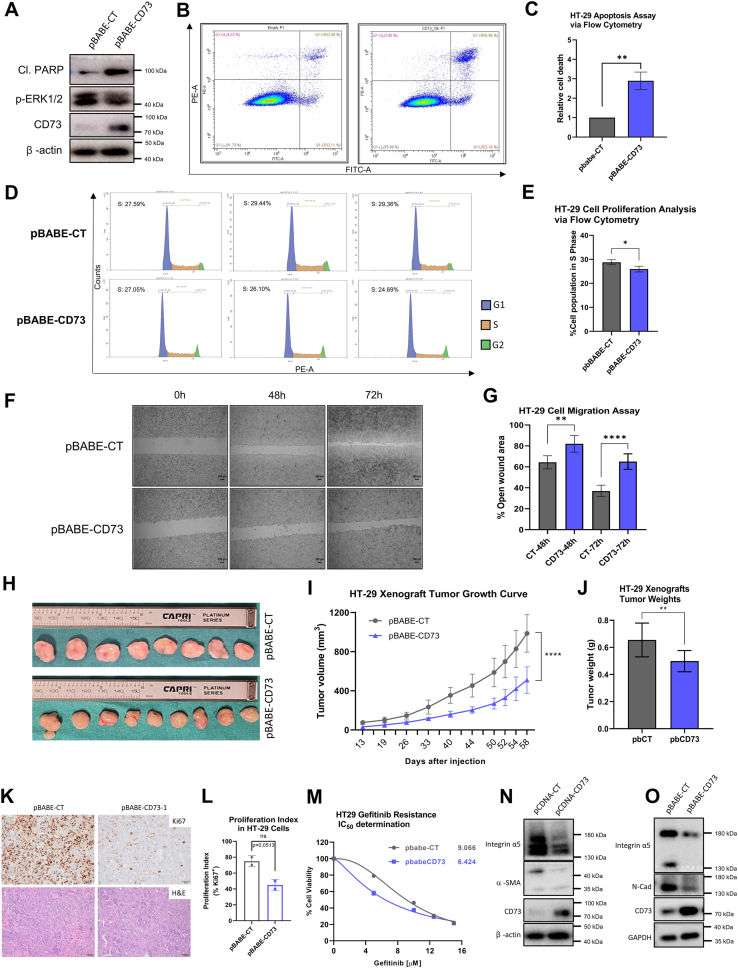
**Overexpression of CD73 in CRC cells displays some reversed phenotypes of CD73 loss of expression.***A* and *C.* CD73 transient overexpression induced apoptosis in HT-29 cells as seen in (*A*) Western blot and flow cytometry *via* (*B*) PI (PE/left axis)-Annexin V (FITC/right axis) staining and (*C*) quantitation of this flow cytometry data. *D* and *J*, CD73 stable transfection reduces cell growth *in vitro*. *D* and *E*, decrease in cell proliferation upon CD73 overexpression is measured *via* flow cytometry with cell cycle analysis (PI-staining). *F* and *G*, impaired cell migration upon CD73 overexpression is quantified *via* wound healing assay. *H* and *J*, CD73 overexpressing cells also form smaller xenografts *in vivo*. *K*, Ki67 and H&E histological stainings in xenograft tumor samples of control and CD73 overexpressing HT-29 cells (scale bar: 100 μm). *L*, proliferation indices based on Ki67 IHC stainings in xenograft tumor samples of control and CD73-overexpressing HT-29 cells, represented as bar graphs. *M*, overexpression of CD73 also sensitizes HT-29 cells to gefitinib. *N* and *O*, EMT signature was also reverted in both transient (N) and stable (*O*) expression of CD73. ∗Western blot panels in (*A* and *N*) were generated from the same lysates for each sample. Therefore, a representative loading for β-actin was used for both panels. IHC, immunohistochemistry; CRC, colorectal cancer.

To test whether the observed effects in CRC cells are specific to CD73 loss, we overexpressed this protein in CRC cells. Ectopic expression of CD73 (CD73-Myc) in HT29 cells clearly induced pronounced cell death which is evidenced by increased cleaved-PARP levels (Fig. 5*A*). To confirm this, HT29 cells that ectopically expressed CD73 were stained with PI-Annexin V. Effectively, an increased apoptotic signal was seen with CD73 overexpressing cells (Fig. 5, *B* and *C*). To check for more effects associated with CD73 overexpression, we created a HT29 cell line which stably expresses CD73(pBABE-CD73) along with a control cell line with empty vector (pBABE-CT). CD73 overexpressing HT29 cells proliferated less (Fig. 5, *D* and *E*) migrated slower (Fig. 5, *F* and *G*) and formed smaller tumors when injected subcutaneously into nude mice (Fig. 5, *H*–*J*) as compared to their controls. We also assessed proliferation in these tumors by Ki67 immunohistochemistry (Fig. 5, *K* and *L*). Although the decrease in proliferation in CD73 overexpressing tumor samples was not statistically significant (*p* = 0.053), the intensity of Ki67 staining appeared noticeably lower compared to control tumors (Fig. 5*K*). Moreover, CD73 overexpressing cells become more sensitive to gefitinib than the control cells (Fig. 5*M*). Both transient and stable CD73 overexpression can partially display inverted EMT signature as seen with some mesenchymal markers such as integrin 5 alpha (integrin α5), N-cadherin, alpha smooth actin (Fig. 5, *N* and *O*). Taken together, it is convincing that CD73 overexpression in CRC cells displays the opposite phenotypes of CD73-deficient cells.

### CD73 expression affects cell fate of CRC cells by multiple cell death inducers

**Figure 6:**
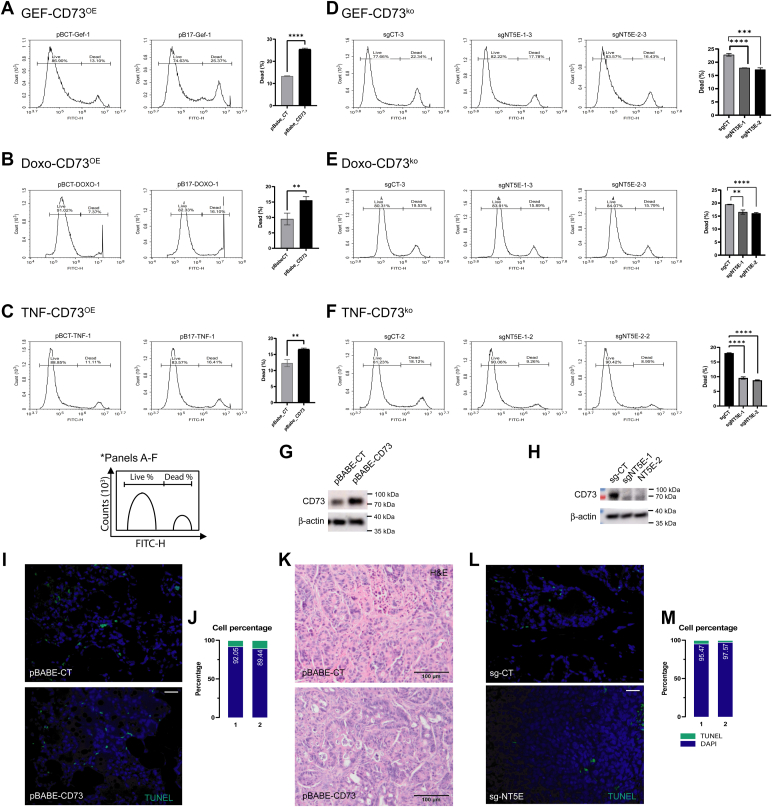
**CD73 expression affects cell fate of CRC cells by multiple cell death inducers.***A* and *C*, CD73 stably expressing HT29 cells (CD73^OE^ by lentiviral pBABE-NT5E) or (*D*–*F*) CD73-deficient HT29 cells (CD73^ko^ by CRISPR/Cas9 (sgNT5E)) were treated with GEF (25 μM, 16 h), Doxorubicin (10 μM, 16 h) and TNF alpha (200 ng/ml, 24 h) respectively. After indicated times for each experiment, cells were harvested and subjected to staining process as described in the Experimental procedures section. Each sample has two peaks of fluorescent equivalent to alive and dead cell populations. There are triplicate for each sample in all experiments and the presented results are presentative of at least two independent repeats. Fluorescent graphs for each experiment are just representative of one sample replicate. *G* and *H*, representative Western blots showing CD73 expression of (*G*) CD73 stably expressing HT29 cells (CD73^OE^) and (*H*) CD73 depleted HT29 cells (CD73^ko^). (*I*) TUNEL staining of xenograft tumor samples from control or CD73 overexpressing HT-29 cells. *J*, quantification of TUNEL-positive *versus* DAPI-only cell percentages in xenografts shown in panel I, represented as bar graph. *K*, H&E staining (magnified from Fig. 5*K*) showing increased number of dead cells in CD73 overexpressing HT-29 (CD73^OE^) xenografts, possibly not undergoing apoptosis. *L*, TUNEL staining of xenograft tumor samples from control or CD73-deficient (CD73^ko^) HT-29 cells. *M*, quantification of TUNEL-positive *versus* DAPI-only cells in xenografts shown in panel L, represented as a bar graph. This scale bar represent 100 μm for all panels. CRC, colorectal cancer.

To further confirm that CD73 intrinsically functions as a tumor suppressor in CRC cells (Fig. 6), we challenged HT29 cells that have stable expression or depletion of CD73 with multiple cell death inducers such as Gefitinib (GEF, Fig. 6, *A* and *D*), Doxorubicin (DOX, Fig. 6, *B* and *E*) and TNF-alpha (Fig. 6, *C* and *F*), which have different induced cell death mechanisms. Cell death was measured by using the membrane integrity-based viability assay (live-dead staining) and analyzed by a flow cytometry. Dead cells were indicated by the population exhibiting high fluorescence intensity, as the reactive dye readily penetrates compromised cell membranes. In contrast, viable cells, which are less impermeable to the dye, were the population displayed lower fluorescence level. Evidence for CD73 expression level are shown in Figure 6, *G* and *H*. With this intensive approach, it was consistently shown that cells with CD73 overexpression were more vulnerable to all tested cell death insults whereas cells with CD73 depletion were protected to some extent from these multiple agents.

Although we typically could not observe decreased cell viability due to CD73 deficiency alone, we were intrigued by the question whether CD73 loss or gain-of-function influences the cell survival in xenografts tumors previously analyzed (Fig. 3, *K*–*P*). To explore this, we assessed apoptotic cell death in HT-29 tumor sections using *in situ* apoptosis detection kit (TUNEL) (Fig. 6, *I*–*M*). These measurements revealed a marked increase in apoptotic cells in CD73 overexpressing tumors (Fig. 6, *I* and *J*) whereas CD73-deficient tumors show fewer apoptotic cells relative to controls (Fig. 6, *L* and *M*). However, the increase in apoptotic rates in CD73 overexpressing tumors did not fully correspond to the extensive cell death (numerous hollow cells) observed in H&E stainings (Fig. 6*K*). This discrepancy suggests that additional, non-apoptotic cell death mechanisms may be involved in CD73-overexpressing cells, potentially explaining our previous observations of reduced cell viability in other experimental settings. These possibilities warrant further investigation in the future with more comprehensive studies.

### CD73 impacts CRC patient survival and EMT in tumors depending on immune cell infiltrates and stroma involvement

**Figure 7:**
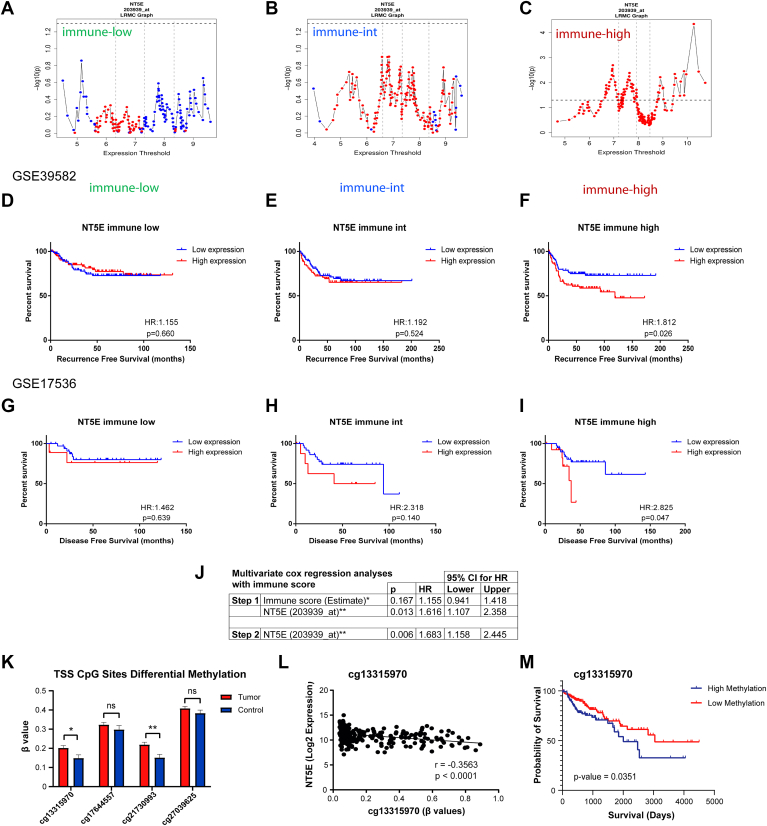
**CD73 impacts CRC patient survival and EMT in tumors depending on immune cell infiltrates and stroma involvement.***A* and *C*, High*NT5E* expression is associated with poor recurrence-free survival in samples only with high immune score (Immune-high) (*C*) yet no such association is observed with immune-intermediate and immune-low groups according to LRMC graphs^#^ (*A* and *B*). *D* and *I*, KM-plots of patients (RFS) according to *NT5E* expression status (high *versus* low) in immune-low, -intermediate and -high groups in *D* and *F*, GSE39582 and (*G*–*I*) GSE17536 datasets. High expression of NT5E is significantly associated with shorter RFS only in the immune-high groups (*I*). 203939_at probeset is used for *NT5E* expression. Median expression is used as a cut-off separately for immune low, int and high groups. *J*, multivariate cox regression analyses with immune score^##^ for RFS. *K* and *M*, *NT5E* promotor methylation is correlated with lower NT5E expression and worse prognosis in TCGA COAD patients. *K*, methylation β levels of the selected CpGs that are located on the transcription start site of the *NT5E* gene in primary tumor (n = 301) and normal (n = 38) colon samples, (∗*p* < 0.05, ∗∗*p* < 0.01, ns: not significant). *L*, scatter plot showing *NT5E* expression and methylation β levels for cg13315970 CpG. Pearson correlation r and *p* values are given. *M*, KM-plot of patients with higher methylation of *NT5E* promotor at cg13315970 had relatively worse survival, (∗*p* < 0.05). ^#^LRMC graph shows gene expression-based cut-offs on the x axis and log-rank *p* values at each specific cut-off on the y axis. The threshold within the interquartile range that gives the lowest log-rank *p* value (6.96) was used to categorize low and high expression groups. For all LRMC graphs: *blue* and *red colors* indicate association with good and poor prognosis, respectively; vertical dashed lines represent 25th percentile, median and 75th percentile values. Horizontal *dashed line* indicates 0.05 *p* value. ^##^ Samples were sorted based on Estimate's immune score. 188, 189 and 189 samples were categorized as immune score low (assigned to 1), intermediate (assigned to 2) and high (assigned to 3), respectively. Assigned values for these categories (1-2-3) were used as continuous variables in multivariate analyses. Patients with available non-zero survival time were included in the analysis. (CI: confidence interval; HR: hazard ratio). CRC, colorectal cancer; KM, Kaplan-Meier; LRMC, log-rank multiple cut-off; RFS, recurrence-free survival.

We sought to answer the question why CD73 expression and CRC prognosis were not coordinated. Since CD73 is known to be expressed in T and B cells, we asked whether the level of immune infiltration in the tumor may contribute to its prognostic relationships. Therefore, we applied the ESTIMATE algorithm which uses gene expression data to give scores reflecting levels of infiltrating immune (Fig. 7, *A*–*C*) and stromal cells (Fig. S4, *A*–*C*). After categorizing CRC tumors into groups with low, intermediate and high immune/stromal scores, we re-analyzed *NT5E*’s prognostic relationships. Our data indicated that NT5E expression was significantly related to unfavorable RFS in immune-high group but not in immune-int and immune-low groups in GSE39582 (Fig. 7, *D*–*F*). Consistently, *NT5E* expression was significantly related to unfavorable disease-free survival in the immune-high group but not in immune-int and immune-low groups in GSE17536 (Fig. 7, *G*–*I*). When tumors were stratified based on stromal scores, high expression of *NT5E* was strongly related to poor RFS and OS in stroma-high group but not in stroma-int and low groups (Fig. S4, *A*–*C*). In parallel to these findings, multivariate analyses (MVA) showed that *NT5E* expression is associated with prognosis (RFS) independent of both immune and stromal groups (Figs. 7*J* and S4*D*). When samples were stratified by the consensus molecular subtypes of CRC (26), *NT5E* was specifically a strong predictor of RFS (cox regression with *NT5E* expression; CMS4: HR:1.443, *p* = 0.003. *p* > 0.05 for CMS1, CMS2 and CMS3) and OS (cox regression with NT5E expression; CMS4: HR:1.341, *p* = 0.011. *p* > 0.05 for CMS1, CMS2 and CMS3) in CMS4 type (Fig. S4, *E* and *F*). These results overall indicate that *NT5E*’s association with poor CRC prognosis can be primarily seen in tumors with a relatively higher stroma content, higher immune infiltration, and the tumors of CMS4 type. In the same line, we have performed additional MVA analyses to identify confounding factors associated with *NT5E* expression and RFS in CRC patients (Tables S1 and S2). These results indicated that high *NT5E* expression was associated with worse prognosis (HR: 1.645; *p* = 0.027), independent of factors like treatment, age, *KRAS* or *BRAF* mutations and tumor location. However, the association remained dependent on MMR status microsatellite instability (MSI) and TNM stage, which were identified as confounding factors. Finally, linear correlation analyses of *NT5E* with EMT markers showed that one probeset of *NT5E* (227486_at) showed positive correlation with almost all mesenchymal markers and negative correlation with epithelial marker *CDH1*, however these correlations got weaker based on r values in immune-intermediate group for almost all markers and were completely lost in immune-low group (Table S3). When stromal groups were evaluated, *NT5E* correlated positively and negatively with mesenchymal and epithelial markers, respectively, in only stroma high tumors (Table S4). *p* values for these analyses can be found in Tables S5 and S6, respectively.

In order to find the reason for the decreased *NT5E* expression in tumor samples, we sought for putative *NT5E* mutations responsible for reduced *NT5E* expression with available sequencing data from TCGA and CCLE (Fig. S5). Although *NT5E* was not showing any mutational hot spot in CRC patients in general, there were a number of cancers with increased deep deletions where deletions were more prominent than amplifications. On the other hand, CCLE CRC cell lines show about 9% alterations half of which were deep deletions while others were missense mutations. Especially, a specific missense mutation (V439 M) which may potentially alter the catalytic cleft of CD73 was found in both CRC patient samples from CCLE and TCGA (Fig. S5, *F* and *J*). There were also other mutations near to catalytic cleft. Although such missense mutations may alter enzymatic function of CD73, they may not be directly relevant to account for reduced NT5E expression in tumors. Moreover, deleterious mutations (frameshift, deep deletion and nonsense) were more common in CRC tumors or cell lines (Fig. S5, *E* and *I*). Hence, we next explored if *NT5E* expression could be epigenetically targeted in CRC tumors by using methylation data available from TCGA-COAD samples in which there were 14 available CpG methylation sites for *NT5E* gene (Fig. 7, *K*–*M*). Among them, we ran correlation analysis between *NT5E* expression and methylation for 4 CpG islands which were located in transcriptional start site near to the *NT5E* promoter and had a mean β value above 0.2. All four CpG islands (cg13315970, cg17644557, cg21730993, cg27039625) showed a negative correlation with NT5E expression (Pearson r < −0.35, *p* < 0.05) (Figs. 7*L*, and S6*A*). 2 of these 4 CpG islands, we noted significantly higher methylation in tumor samples compared to adjacent normal colon tissue (Fig. 7*K*). High *NT5E* promoter methylation at cg13315970 was significantly associated with worse prognosis in CRC patients (Fig. 7*M*), while methylation of all other CpG island were near significant (Fig. S6*B*). Collectively, these results strengthen our hypothesis that *NT5E* gene may be epigenetically regulated to reduce its transcriptional level in CRC tumors.

## Discussion

### Correlation between CD73 expression and CRC disease

We aimed to provide a general examination of the most frequently used cell lines in CRC as available data on CD73/*NT5E* expression in CRC cell lines was still limited. Indeed, CD73 was strongly expressed in tested CRC cell lines. We found that CD73 was not detected in RKO cells as observed by another group (27) despite using different CD73 antibodies. Of important note, SW620 and SW480 cell lines were derived from the same patient but they had contrasting CD73 levels. The reason may lie in the fact that the SW620 cell line was derived from a metastatic tumor sample. This finding was supported by research in which CD73 expression in SW48 cells was much reduced when cells were modified to metastasize efficiently into the liver after intrasplenic injection, as compared to the parental SW48 (28). It was deduced that CD73 decline may involve the mechanism of metastasis of this cell line.

Data on CD73 expression in CRC patients were not ample. A two cohort study in 2012 reported that about 50% of samples had high *NT5E* expression and were associated with lower overall survival (29). A more recent study in human rectal adenocarcinoma; however, showed that patients with high *NT5E* expression had significantly better clinical outcome. Upregulated *NT5E* expression is found in both tumoral and stromal compartments, but concomitantly high stromal CD73 would lead to improved outcomes (16).

Our data also showed CD73 as a poor prognostic marker in the immune and stroma high groups and independence from both immune and stromal groups in MVA, suggesting that a tumor microenvironment rich in immune and stromal cells might be contributing to CD73’s prognostic role. Whereas in immune and stroma low tumors, CD73 lost its prognostic significance. We also showed that the expression of *NT5E* from inflammatory cells was not prominent compared to other stromal cell types in CRC tumors. Since stroma-rich tumors harbor distinct molecular characteristics compared to other tumor types (30) as shown in CMS subtypes (26). NT5E expression may be an indicator of poor prognosis in more aggressive sub-groups of CRCs which is in line with the fact that HIF-1α which is highly expressed in stroma rich hypoxic TME can enhance the expression of CD73 (31). This is supported by its most significant prognostic relationship observed in the CMS4 subtype which is considered “mesenchymal and stroma rich” and is the subtype with the poorest survival among consensus molecular subtypes of CRC. Additional analysis with MVA to determine confounding factors associated with NT5E related prognosis in CRC patients support previous observations that, in tumors with MSI (likely highly immunogenic and immune-infiltrated) and in advanced TNM stages (due to lymph node involvement and increased immune cell infiltration), high NT5E expression is associated with worse prognosis. Further research is needed to enlighten the underlying molecular mechanism for these relationships.

### Intrinsic roles of CD73 in CRC

While CD73 is widely acknowledged as a tumor promoting protein and its inhibition of this effect would be beneficial for CRC treatment (Yang *et al.* 2021), there are challenges and limitations in the application of immunotherapies to CRC patients (32). It is important to bear in mind that CRC is a heterogenous disease developed with the contribution of many genetic and epigenetic factors. Furthermore, CRC has a deep link with inflammation and the interaction of cancer cells with the TME is pivotal in tumorigenesis. TME is constituted of three main cell types: cancer-associated fibroblasts, vascular cells and infiltrating immune cells (1). In one study, CD73 expression in certain subsets of fibroblasts rather than tumor cells was found to be important for the oncogenic effects of CD73 in murine colorectal tumorigenesis (33). These findings supported previous observations with mesenchymal stem cells expressing CD73 in gastric cancer (34). Therefore, the oncogenic role of CD73 in the TME may be primarily dependent on fibroblasts. Moreover, these studies put forward that tumor cell specific CD73 expression can be dispensable in inducing immunotolerance and immune evasion. Impact of CD73 expression on tumor progression and survival is likely influenced by the degree of immune and stromal cell infiltration, as well as the specific cellular context within the tumor microenvironment. This strengthens the notion that CD73 expression in colonocytes may be important for other functions as we explained in this work. The intrinsic roles of CD73 in each cell type need to be carefully dissected so that the targeted therapy could be more precise and effective.

To address the intrinsic roles of CD73 in cancer cells of CRC, we generated several CD73-deficient CRC cell lines to confirm its effects. Indeed, our results were very consistent, raising the reliability of this research. All CD73-deficient CRC cell lines had increased proliferative capacities in both *in vitro* (including 2D and 3D settings with polyHEMA) and *in vivo*. We also demonstrated that these cells gained motility through improved migration and invasion. Supporting the role of CD73 in cell adhesion/migration, it was shown that reduced expression of CD73 at the cell surface also increased migration in astrocytes as measured by the wound healing assay (35). Moreover, this effect was dependent on CD73 enzymatic activity. To explain this tumor suppressive role of CD73, we presented here that CD73 deficiency involves EMT induction. Vice versa, our lab-generated-GR CRC cell lines, in which increased EMT is usually a signature for drug resistance, also harbor much less CD73 protein. These findings were in line with the aforementioned fact that metastatic cell lines such as SW620 or the aforementioned liver metastatically engineered SW48LM2 cell subline (28).

A recent report using colitis associated carcinoma model showed that CD73 promoted tumor development as evidenced by reduced tumor burden in mice after treating with an CD73 pharmacologic inhibitor, APCP, supporting an oncogenic role for CD73 in mouse tumorigenesis (36). At first glance these results are in stark contrast with our findings. However, since colitis-associated carcinoma is a good model of immune infiltrated tumors that does not conflict with our bioinformatic analyses, it is expected that the oncogenic role of CD73 will be pronounced in highly immune-infiltrated tumors.

In a previous report it was shown that CD73 facilitates EMT progression and suggested that it promotes early steps of tumor progression, possibly through facilitating epithelial-mesenchymal transition in triple-negative breast cancer (37). In line with that, our bioinformatic analyses showed that CD73 is positively correlated with mesenchymal markers rather strongly in the immune and stroma high group and its related to poor prognosis in immune-stroma high tumors. Therefore, EMT may be an important mechanism underlying the tumor-promoting roles of CD73.

Furthermore, the single cell RNA-Seq data showed the highest expression of CD73 in endothelial cells and CAFs in 4 out of 6 donors analyzed (based on two probesets with the highest mean). Therefore, either expression of CD73 from stromal/immune cells may have dominant roles in patient outcome or it may be a potential marker of poor prognosis in stroma-immune rich CRC subgroups. The fact that correlation with EMT markers vary dramatically for different probesets of *NT5E* may suggest an involvement of multiple transcript variants in the prognostic role of CD73. Therefore, the expression of different CD73 variants from various cellular sources may contribute to downstream molecular mechanisms. Analysis of transcriptomic data overall showed that prognostic and molecular relationships of CD73 change dramatically with changing immune and stromal cell levels in the tumor microenvironment *in silico*, which may explain its contrasting tumor-suppressor-like relationships *in vitro* compared to associations with poor prognosis in patient tumors. Our findings also showed that expression and methylation levels of *NT5E* were negatively correlated and that the primary colon tumors have higher methylation compared to adjacent normal tissue. Previous studies also showed such a negative correlation in pancreatic cancer (38). Higher methylation levels were associated with poor prognosis, which can be interpreted as the methylation mediated suppression of *NT5E* expression may be related to poor prognosis supporting anti-tumor effects observed *in vitro* and *in vivo*.

Some previous reports supported our surprising findings of the tumor suppressive role of CD73 in CRC. In endometrial carcinoma, CD73 expression was shown to be markedly reduced in poorly differentiated and advanced-stage as compared to its levels in normal endometrium and low-grade tumors (14). In murine models, CD73 loss was shown to be responsible for a loss of endometrial epithelial barrier function (39). CD73 was also found to enhance endothelial barrier function only in blood vessel formation (40). Another study put forward that loss of CD73 in endometrial cancer may change tumor suppressor effects of TGFβ to tumor promoter by the loss of epithelial integrity and increased invasiveness (41). In this view, there may be similar mechanisms related to CD73 expression and intestinal barrier function in colorectal tumors. Inhibition of CD73 may lead to loss of epithelial integrity, favoring migration and invasion of cancer cells and maintaining a chronic inflammatory microenvironment due to invading bacteria. Collectively, differential cell type specific functions of CD73 in tumorigenesis of various cancers are valuable subjects to elaborate (42).

As a result of our analyses, CD73 in tumor cells exhibited many hallmarks associated with tumor suppressor genes across multiple experimental approaches in this study. CD73-deficient cells showed increased proliferation, EMT, migration, and survival, whereas these phenotypes were successfully reversed in CD73-overexpressing cells. Notably, histological examinations of xenograft tumor samples confirmed the increased proliferation and reduced cell death observed in CD73-deficient cells. Tumor phenotypes evaluated through *in silico*, *in vitro*, and *in vivo* models collectively suggest that CD73 may function as a context-dependent tumor suppressor. However, given the complexity of the tumor microenvironment and the potential cell type–specific roles of CD73 in the tumor stroma, more advanced models will be required to fully dissect its context-dependent functions in CRC development.

Collectively, our findings, together with recent literature, suggest that CD73 exerts context-dependent roles in both immune evasion and cell viability. The mechanisms driving CD73 overexpression likely depend on tumor immunogenicity, which can be influenced by factors such as genotoxic stress, MSI, high apoptotic burden, extracellular ATP accumulation, and the upregulation of cytokines and chemokines within the tumor microenvironment (43). These signals may enhance immune cell infiltration and thereby strengthen anti-tumor immunity (44). In response, numerous mechanisms—including immune checkpoint signaling, adenosine signaling, and cytokine pathways that activate M2/Th2 or Treg cells—are upregulated by both tumor and stromal cells to promote immunotolerance. Among these pathways, CD73 upregulation is particularly important for converting ATP to adenosine, which suppresses effector and cytotoxic T-cell activity (45, 46). Consequently, CD73 overexpression becomes critical for tumor cell survival under immune attack (47). Conversely, in tumors with low immune infiltration and minimal stromal support, high CD73 expression may not confer a survival advantage due to the lack of heterotypic interactions (48). In fact, our results indicate that CD73 overexpression in tumor cells alone decreases their viability and survival under intratumorigenic stress. Tumor cells deprived of heterotypic interactions and exhibiting high CD73 expression may become sensitized to death signals by mechanisms yet to be identified. Therefore, CD73’s role in colorectal tumors depends on the complex interactions between tumor cells and the stroma, underscoring its context-dependent function.

In conclusion, the role of CD73 in cancer is complex, as cancer itself is a tissue-specific disease. For this very reason, a "one size fits all" strategy in therapy does not guarantee effective treatment. In our case, CD73 functions as a tumor suppressor in CRC, depending on the stages, localization, and even the composition of the TME.

## Experimental procedures

### Cell culture

All mammalian cell lines were cultured at 37 °C in a humidified cell incubator with 5% CO_2_. HT29 (RRID: CVCL_0320), SW480 (RRID: CVCL_0546), RKO (RRID: CVCL_0504), SW620 (RRID: CVCL_0547), and SW48 (RRID: CVCL_1724) were maintained in DMEM (Capricorn Scientific) supplemented with 10% Fetal Bovine Serum (FBS) and 1% L-glutamine; DLD-1 (RRID: CVCL_0248), and HCT116 (RRID: CVCL_0291) were maintained in McCoy’s 5A (Capricorn Scientific) supplemented with 5% FBS and 8% FBS, respectively. All the cell lines were obtained from ATCC or LGC Standards. Authenticity of the cells is regularly checked by STR profiling. Cell lines used in this study were regularly checked to ensure the mycoplasma-free cells by the use of MycoAlert *mycoplasma* detection kit.

#### Generation of CD73-deficient cell lines by CRISPR-Cas9

CD73 is encoded by the *NT5E* gene. Various bioinformatic tools were used to choose three best commonly suggested gRNA sequences as listed: sgControl: GCACTACCAGAGCTAACTCA; sgNT5E-1: GCAGCACGTTGGGTTCGGCG; sgNT5E-2: CTATGTGTCCCCGAGCCGCG; sgNT5E-3: ATCTGGTTCACCGTGTACAA. Annealed oligos of the designed gRNA were cloned into the lentiCRISPRv2 vector (RRID: Addgene_98290). Constructs with correct sequences were used to generate knockout cell lines by lentiviral transfection procedure. Depleted cells were selected with 3-6 μg/ml puromycin (Invivogen).

#### Plasmid vectors and overexpression studies

pBABE vector (RRID: Addgene_21836) and pCX-TRI-2A vector (RRID: Addgene_74674) were obtained from Addgene. h*NT5E* gene is cut from pCX-TRI-2A vector and cloned into pCDNA3 or pBABE vectors for the generation of overexpression plasmids. These constructs were transfected *via* Transfectin (Bio-Rad) or Lipofectamine 2000 (Thermo Fisher Scientific) for transient or constitutive expression of *NT5E* in CRC cell lines, respectively.

#### Generation of gefitinib-resistant cell lines

To generate GR cell lines, cells were exposed to increasing drug concentrations over the time, starting with a concentration slightly below their IC_50_ values. GR DLD1, HT29 cells were obtained roughly after a year when their IC_50_ had reached approximately twice more than their initial values (49). IC_50_ for drugs was determined by cell viability assay with Sulforhodamine B. The detailed protocol is found in the supplementary information.

### Animal studies

6 to 8 weeks old male athymic nude mice (RRID: IMSR_JAX:002019) were used for cell line-derived xenograft models. All the mice used in these experiments were breed and obtained from the Department of MBG Experimental Animal Facility. Cells were counted and prepared in 200 μl PBS for subcutaneous injection. Tumor volumes were measured by digital caliper and recorded 3 times a week, using the following formula L x W^2^ (L: length, W: width). Mice were sacrificed when tumors approximately reached 15 mm in diameter or 1500 mm^3^ in volume. All experimental procedures are approved by the Animal Experiments Local Ethics Committee (HADYEK) at Bilkent University with decision no. 2016/24 (later updated in 2018).

### Cellular and biochemical assays

Detailed information on all relevant cellular and biochemical analyses can be found in the supplementary information.

### Histological analyses

Formalin-fixed, paraffin-embedded tissue sections were stained immunohistochemically using a polyclonal anti-Ki-67 antibody (Thermo Fisher Scientific, RM9106-SO; 1:100). Sections were deparaffinized by incubation at 60 °C overnight, then dipped twice in xylene for 10 min each and rehydrated through a graded ethanol series. Antigen retrieval was performed in EDTA buffer (pH 8.0) using microwave heating for 10 min. Endogenous peroxidase activity was blocked with 7% hydrogen peroxide in 80% methanol. After blocking, the primary antibody was applied for 1 h at room temperature. Sections were then incubated with a biotinylated secondary antibody and streptavidin-HRP complex (Thermo Fisher Scientific, Lab Vision, anti-polyvalent HRP), each for 30 min. Immunoreactivity was visualized using 3, 3′-diaminobenzidine.

For the quantification of apoptosis in xenograft tumors, formalin-fixed, paraffin-embedded tissue sections were stained with *in situ* apoptosis detection kit (MK500, Takara) according to manufacturer’s protocol. After deparaffinization, each section was treated with Proteinase K and incubated at room temperature for 15 min, followed by a single PBS wash. The labeling reaction mixture (consisting of TdT enzyme and labeling buffer) was applied to each slide which are then incubated at 37 °C in a humidified and dark environment for 60 min. After incubation, the reaction was stopped by washing the slides three times with PBS. Finally, slides were covered with coverslip by the use of Prolong Gold Antifade Mountant with DAPI (Thermo Fisher Scientific). Stained tissue sections were visualized using Zeiss (Axio Imager Z2) fluorescence microscope.

### Bioinformatic analyses

Detailed information on all relevant bioinformatic analyses can be found in the supplementary information.

### Statistical analysis

In bar graphs, data were expressed as mean ± SD. Statistical differences were evaluated by Student’s *t* test for bar graphs, one-way ANOVA test for evaluating differences in IC_50_ values and two-way ANOVA for evaluating differences in tumor growth graphs were used in GraphPad Prism 8 software (https://www.graphpad.com/). In these analyses, *p* values ≤ 0.05, covering 95% confidence intervals, were considered significant. All other bioinformatic analyses with human patient data and related statistics were explained in the relevant parts within supplementary information.

## Ethics Statement

All the experimental protocols related to animal studies are reviewed and approved by the Bilkent University Animal Ethics Committee. Registry number for the protocol: #2016/24.

## Data availability statement

The datasets presented in this study can be found in publicly available repositories. The names of the repositories and accession numbers can be found in the following: Microarray: GSE39582 and GSE17536; FACS-sorted microarray: GSE39396; Healthy, adjacent and tumor tissue array: GSE44076; Single cell RNA-Seq: GSE178318, and GSE178341 datasets were obtained from GEO database (https://www.ncbi.nlm.nih.gov/geo/query/acc.cgi); clinical data was obtained from Array Express (http://www.ebi.ac.uk/arrayexpress); CMS subtypes were obtained from https//www.synapse.org. All the details of the data analysis can be found in the supplementary materials. Mutation and copy number alteration data was obtained from cBioPortal (https://www.cbioportal.org/). TCGA COAD RNAseq and methylation data was utilized through TCGAbiolinks R package.

## Supporting information

This article contains supporting information.

## Conflict of interest

The authors declare that they have no conflicts of interest with the contents of this article.
