# Supplementary material for: Purinergic ecto-enzyme CD73 is a context-dependent tumor suppressor in colorectal cancer
Source: J Biol Chem. 2025 Oct 27;301(12):110864. doi: 10.1016/j.jbc.2025.110864 (PMC12666832; doi:10.1016/j.jbc.2025.110864)
Supplement: Supplementary Information [file mmc1.pdf]

Supplementary Information

Purinergic Ecto-Enzyme CD73 is a Context-Dependent Tumor Suppressor in Colorectal Cancer

Tieu Lan Chau[1], Ümran Borucu[1*], Beste Uygur[1*], Ahmet Göktuğ Özkurt[1*], Eylül Kılıç[1], Aynur Işık[2], Ayşe Dila Gemalmayan[1], Caglar Cekic[1], Aytekin Akyol[2,3], Seçil Demirkol Canlı[4,5], Serkan İsmail Göktuna[1]

[1]Department of Molecular Biology and Genetics, Bilkent University, Ankara, Türkiye

[2]Transgenic Animal Technologies Research and Application Center, Hacettepe University, Ankara, Türkiye

[3]Department of Pathology, Hacettepe University Faculty of Medicine, Ankara, Türkiye

[4]Molecular Pathology Application and Research Center, Hacettepe University, Ankara, Türkiye

[5]Tumor Pathology, Cancer Institute, Hacettepe University, Ankara, Türkiye

[*]These authors have contributed equally to this work

Correspondence:

Serkan İsmail Göktuna; serkan.goktuna@bilkent.edu.tr
ORCID ID: 0000-0001-6169-768X

Contents of Supplementary Information:

1 Supplementary Materials and Methods

2 Supplementary Figures

3 Supplementary Tables

4 References

1 Supplementary Materials and Methods

1.1 In vitro Analyses

1.1.1 Western Blots

Protein extraction was performed with RIPA cell lysis buffer (150mM NaCl, 5mM EDTA pH 8.0, 50mM Tris pH 7.5, 1% NP-40, 0.5% sodium deoxycholate, 1% SDS) supplemented with protease and phosphatase inhibitor cocktails. After centrifugation, protein lysate concentration was determined by the BCA kit (ThermoFisher). Boiled protein samples were run in SDS-PAGE and transferred to PVDF membranes (Advansta) via a wet transfer system. Membranes were next blocked with 10% skim milk for 30 minutes at RT and washed with TBS-T before incubation with primary antibody followed by secondary antibody. Membranes were revealed using ECL Western Blotting Substrate (Thermofisher) with the Amersham Imager 600 machine (GE Healthcare Life Sciences).

1.1.2 Quantitative PCR

RNA extraction was performed with E.Z.N.A. Total RNA Kit I (Omega Bio-Tek). iScript cDNA Synthesis Kit (Bio-Rad) was used to generate cDNA which were then diluted and subjected to quantitative PCR using SYBR green (LightCycler® 480 SYBR Green I Master; Roche) and The LightCycler® 480 System (Roche).

1.1.3 Flow Cytometry

All analyses were carried out with the CytoFlex (Beckmann Coulter) cytometer. For surface staining, cells were trypsinized, washed, then incubated with F_C block for 15 minutes on ice before primary antibody incubation. Cells were subsequently washed and resuspended in 200µl of FACS buffer to be analyzed. Flow cytometry-based cell cycle analysis was performed through propidium iodide (PI) and CytoExpert software was used to analyze cell cycle distribution.

For live-dead staining, LIVE/DEAD Fixable Dead Cell Stain Kit (ThermoFisher) was employed. Dye preparation was performed according to manufacturer's instructions. Cells were harvested and washed with PBS. Subsequently, cell density was adjusted to 1×10^6 cells in 1 mL PBS. For each replicate, 1 uL of fluorescent reactive dye was added and incubated for 30 minutes on ice in dark. Following incubation, cells were washed twice with PBS with 1% bovine serum albumin. Finally, cells were resuspended in 1 mL PBS with 1% BSA and acquired in the FITC channel at 488 nm excitation and 530/30 detection of flow cytometer (NovoCyte 3000). Gating was established to eliminate the signals originating from cell debris and to select populations corresponding exclusively to live and dead cells (within 50,000 events).

1.1.4 Cell proliferation assay

Cells were seeded in 96-well plates and grown under normal culture conditions. CellTiter-Glo® Luminescent Cell Viability Assay was performed at indicated time and according to the protocol provided by the manufacturer. Luminescence was then read by Synergy HT Microplate Reader.

1.1.5 Anchorage independent cell growth assay

To be able to demonstrate anchorage-independent cell growth, poly-2-hydroxyethyl methacrylate (polyHEMA) coated plates were utilized. First a 120mg/ml stock polyHEMA was fully dissolved in 95% Ethanol. Next 96-well plates were coated twice with 50µL of diluted polyHEMA 40mg/ml each time to get a total amount of polyHEMA 4mg/well. Keep the coated plates in a non-humid incubator at 37°C until the coatings are completely dry for each layer. Cells were next seeded into these plates and followed up over 1 week. Images of colonies were captured under inverted microscope on different days.

1.1.6 Colony formation assay

Cells were seeded in low numbers so that they can grow as separated colonies. Medium was renewed every 2-3 days until 2-3 weeks. Cell plates were washed with PBS, fixed with ice-cold methanol for 10 minutes, and stained by 0.5% crystal violet. Images were quantified by the ImageJ program.

1.1.7 Wound healing and cell invasion assays

Cells were seeded to reach 100% confluency the next day; the gap was created by using a 200µl tip to scratch. Reduced serum was replaced to distinguish migratory ability from their proliferation. The images were captured at the starting point and at the different time points to follow the gap closure which was then quantified by the Image J software. Open wound area was calculated by the percentage of final gap area to the initial gap area.

For invasion assay, Transwells (Corning) were coated with 50µl of 0.2mg/ml Matrigel (Corning) and left in the 37°C incubator for 5-6 hours. Next, cells resuspended in medium with 1% FBS were loaded, 750µl medium supplemented with 20% FBS were added to surround the transwells. At the end of the experiment, transwells were fixed, washed and stained with Giemsa. Transwells were left to dry completely before processing the images.

1.1.8 IC_{50} determination

IC_{50} for drugs was determined by cell viability assay with Sulforhodamine B (SRB). Cells were seeded in a 96-well plate and treated with drugs the next day. After 72 hours, old media were replaced by 120µl of the new ones and 80µl of 25% trichloroacetic acid (TCA) was added to each well. The plates were next incubated at 4°C at least an hour for fixation. After incubation time, the mixture was removed and the wells were washed multiple times with ddH_2O. Once excessive water was completely removed from the wells, 50µl of 0.4% SRB was added to each well to stain the cells for 30 minutes at RT in the dark. Unbound stain was washed off by 1% acetic acid and the plates were left for air-dry. Finally, 150µl of 10mM Tris-Base pH10 was added to destain and the plates were read at 564nm with Synergy HT Microplate Reader. IC_{50} was calculated with GraphPad Prism 8 (GraphPad Software, CA, USA).

1.2 Bioinformatic Analyses

1.2.1 Microarray data processing

CEL files of 566 and 177 colorectal tumors in GSE39582 (Marisa *et al.* 2013) and GSE17536 (Smith *et al.* 2010) datasets were downloaded from NCBI GEO database (https://www.ncbi.nlm.nih.gov/geo/query/acc.cgi) and RMA normalized using "affy" package in R

Bioconductor (Gautier *et al.* 2004). Clinical data was obtained from Array Express (http://www.ebi.ac.uk/arrayexpress). Microarray data from FACS separated CRC tumors were obtained from GSE39396 dataset (Calon *et al.* 2012) and RMA normalized using Applied Biosystem Transcriptome Analyses Console (TAC) Software. For analysis of NT5E expression, microarray dataset which contains paired primary colon tumor (n=98), tumor adjacent normal mucosa (n=98) and colon mucosa from healthy donors (n=50) gene expression profiles in GSE44076 were obtained from NCBI GEO database. RMA normalization was performed using "affy" R package. Mean of 3 probesets of NT5E was used for the scatter plot (11719174_a_at, 11744681_a_at, 11755207_a_at). The probe sequences for Affymetrix Human Genome U133 Plus 2.0 and Affymetrix Human Genome U219 Arrays can be accessed through the Bioconductor packages "hgu133plus2probe" and "hgu219probe", respectively.

1.2.2 Generation of CRC tumor sub-groups based on other methods

Stromal and immune scores of CRC tumors were generated based on transcriptomic data utilizing the "estimate" package in R Bioconductor (https://bioinformatics.mdanderson.org/estimate/) (Yoshihara *et al.* 2013). Samples were divided into three groups of almost equal sizes based on stromal and immune groups separately. The groups were named as "high", "int" and "low" referring to high, intermediate and low scores, respectively. CMS subtypes for GSE39582 were downloaded from www.synapse.org, from file 'clinical_molecular_public_all.txt'. Samples assigned to CMS 1, CMS2, CMS3, or CMS4 were included in the analyses.

In CIBERSORT, the absolute score reflects the estimated absolute proportion of each cell type in a sample, providing a measure of cell abundance. To identify abundances of infiltrating immune cells depending on CD73 expression in patient samples (GSE39582 dataset), we have used a similar methodology as described previously (Chen et al. 2016). Briefly, CIBERSORT analysis run by the use of "CIBERSORT" package in R Bioconductor (Newman et al. 2015). For the correlation analysis, we have calculated Pearson correlation for CIBERSORT data vs. CD73 expression (in 4 different probesets) as described in "correlation analyses" (Supplementary 1.2.7).

1.2.3 Single cell RNA-seq Data Processing

For the scRNA-seq analysis of colon cancer, expression matrix and annotation files of GSE178318, and GSE178341 datasets are downloaded from GEO database. Single cell RNA-seq analysis was performed by using Seurat R package (Hao *et al.* 2021). Among 6 CRC primary tumors available in GSE178318, 3 treatment naïve tumors were used for the analysis. GSE178318 data was filtered to exclude the cells which have high mitochondrial gene expression (>15%) and number of unique genes detected in each cell (nFeature) higher than 6000 or lower than 500. For the normalization and scaling, standard Seurat workflow was followed. For the clustering and the UMAP visualization, first 15 dimensions were used with the resolution of 0.1. For the identification of the cell types in GSE178318, markers that are provided by the authors were used (Che *et al.* 2021). Moreover, DoubletFinder (McGinnis *et al.* 2019) R package was used for the removal of the doublets in GSE178318 dataset. For GSE178341 (n=62), pre-processed count matrixes were used with the metadata of the Seurat objects provided by the authors (Pelka *et al.* 2021). Clustering and UMAP visualization were performed as described for GSE178318.

1.2.4 Analysis of NT5E expression in tumor and normal tissues in TCGA-COAD

For comparison of NT5E expression in primary solid tumor vs normal colon, RNAseq data of 456 tumors and 41 adjacent normal tissues of TCGA-COAD dataset were obtained via TCGAbiolinks package (Colaprico *et al.* 2016). For the analysis of paired samples, 41 tumor and adjacent normal colon samples were extracted. Normalization and vst transformation were performed by using DESeq2 R package. Welch's t-test and paired t-test were used for comparison of expression in tumor and normal samples for unpaired and paired data, respectively. Moreover, NT5E mutations and copy number alterations were analyzed through cBioPortal platform (https://www.cbioportal.org/) (Gao *et al.* 2013).

1.2.5 Analysis of TCGA COAD Methylation and Gene Expression Data

Gene expression quantification and methylation data of colorectal cancer tumors were obtained from the TCGA-COAD project using TCGAbiolinks R package (Colaprico *et al.* 2016). Formalin-fixed, paraffin embedded (FFPE) tissues were removed from both datasets. Patients with both methylation and expression data were used for the analysis of correlation between NT5E expression and methylation. For samples run in duplicates, expression or methylation data with higher total counts or β values were used. Raw RNA-seq counts 285 primary tumors were normalized and vst transformed using the DESeq2 R package (Love *et al.* 2014). β values were available for 14 CpG islands within NT5E gene for these 285 patients. 4 out of 14 CpG islands fulfilling the following criteria; 1. Located at the transcription start site (TSS) of NT5E gene, 2. Mean β value higher than 0.2 were included in the analysis of linear correlation between expression and methylation levels. Methylation data from 301 primary tumor and 38 adjacent normal colon tissue obtained from TCGA-COAD data were utilized for the comparison of NT5E methylation levels between tumor and normal.

1.2.6 Survival analyses

Log rank multiple cut-off graphs were generated as described previously (Demirkol *et al.* 2017). Briefly, prognostic differences were evaluated based on log-rank p values plotted on the y axis, comparing the "high" and "low" expression groups generated based on all possible cut-offs shown on the x axis. Red and blue colors indicate association of high NT5E expression with poor and good prognosis, respectively. Vertical dashed lines represent 25 percentile, median and 75th percentile values. Horizontal dashed line indicates 0.05 p value. Kaplan Meier graphs were generated using GraphPad Prism 6, for Windows (GraphPad Software, CA, USA). Multivariate Cox regression analyses (MVA) were performed using IBM SPSS Statistics v.23.0 for Windows (IBM Corp., NY, USA). For prognostic analyses NT5E probeset "203939_at" is used which had the highest mean expression among NT5E probesets in both GSE39582 and GSE17536. Samples with a non-zero survival value were included in all prognostic analyses. For the survival analysis of patients with CpG island methylation in NT5E promotor, cut-offs which had the lowest p value within 15th and 85th percentiles were used for the generation of Kaplan Meier graphs.

1.2.7 Correlation analyses

Linear correlation of CD73 expression with the expression of EMT markers were assessed using Pearson function in Microsoft Excel (2013). Among multiple probesets of EMT marker genes, the probesets with highest variation were used.

1.2.8 Human Protein Atlas Data

Human normal or tumor tissue NT5E IHC results were obtained from the Human Protein Atlas (HPA) data which is publicly available at https://www.proteinatlas.org/. The antibody used in these stainings was described as: “Anti-CD73 (HPA017357) antibody (Sigma) produced in rabbit, a Prestige Antibody, is developed and validated by the Human Protein Atlas (HPA) project. Each antibody is tested by immunohistochemistry against hundreds of normal and disease tissues (Haun et al. 2015)”. Patient IDs: healthy (3266), CRC-1 (2001), and CRC-2 (2096).

1.2.9 UALCAN Platform (CPTAC Data)

Publicly available MS-based human protein expression data from Clinical Proteomic Tumor Analysis Consortium (CPTAC) is analyzed by using UALCAN platform to obtain protein expression data. We have obtained results from UALCAN website (https://ualcan.path.uab.edu/cgi-bin/CPTAC-Result-mod.pl?genenam=NT5E&ctype=Colon). We endorse the use of UALCAN for proteomic analyses (Chandrashekar et al. 2022).

2 Supplementary Figures

Figure S1

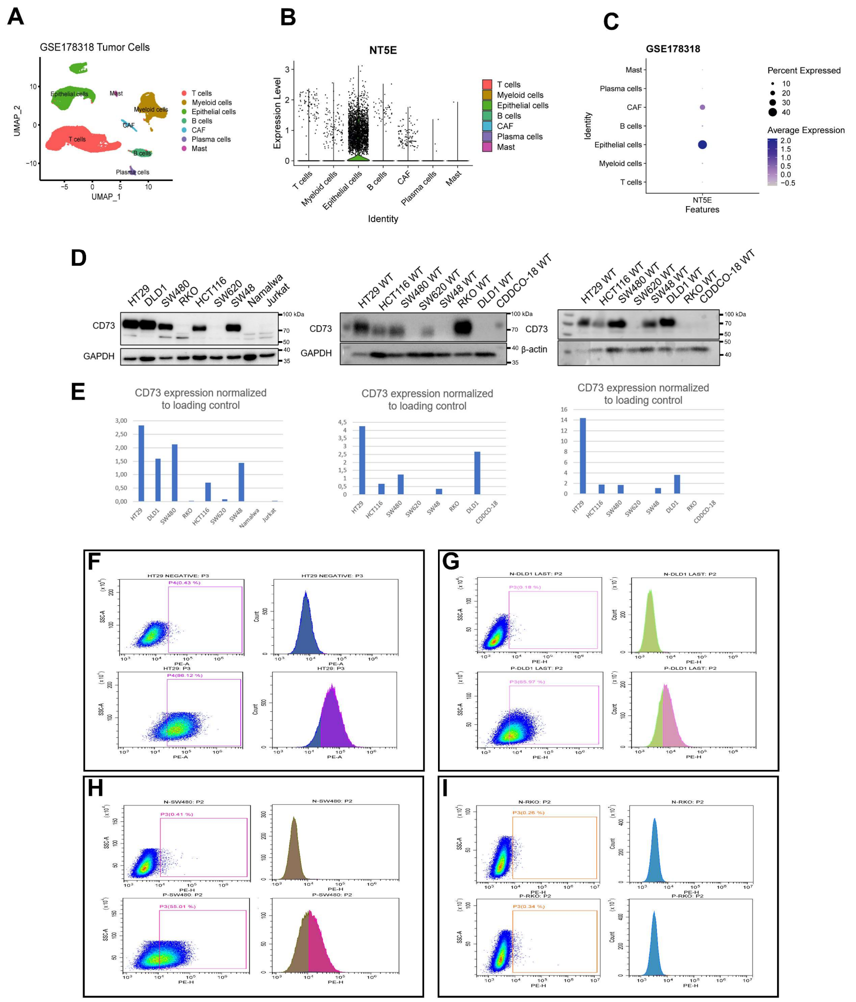

Supplementary Figure 1. Supplementary information for Figure 2. (A-C) UMAP based visualization of GSE178318 dataset which contain 6 primary tumor samples of CRC patients. Clusters were either colored according to **(A)** different cell populations or **(B)** NT5E expression levels. **(C)** Average NT5E expression in each cluster in UMAP were also visualized via dot plots. (**D**) Western blot analysis of CD73 expression from three independent experiments (including results from main Figure 2 G) using CRC cell line extracts, compared to non-malignant colon epithelial cells. **(E)** Quantification of CD73 expression normalized to the loading control, presented as bar graphs. **(F)** HT-29, **(G)** DLD-1, **(H)** SW480 and **(I)** RKO cells CD73 cell surface expression profiles related to the graphs presented in Figure 1I and 1J. HT-29, DLD-1 and SW480 cell lines are exemplified since most of the experiments were carried in these cells. RKO is included for comparison since there was no detectable CD73 expression in these cells. In each panel, upper two graphs show the negative stainings while lower two graphs show positive stainings for CD73. For all FACS analysis, positive cells (within P3 gating, dot plots) indicated with a different color than the negative cell populations (dendograms).

Figure S2

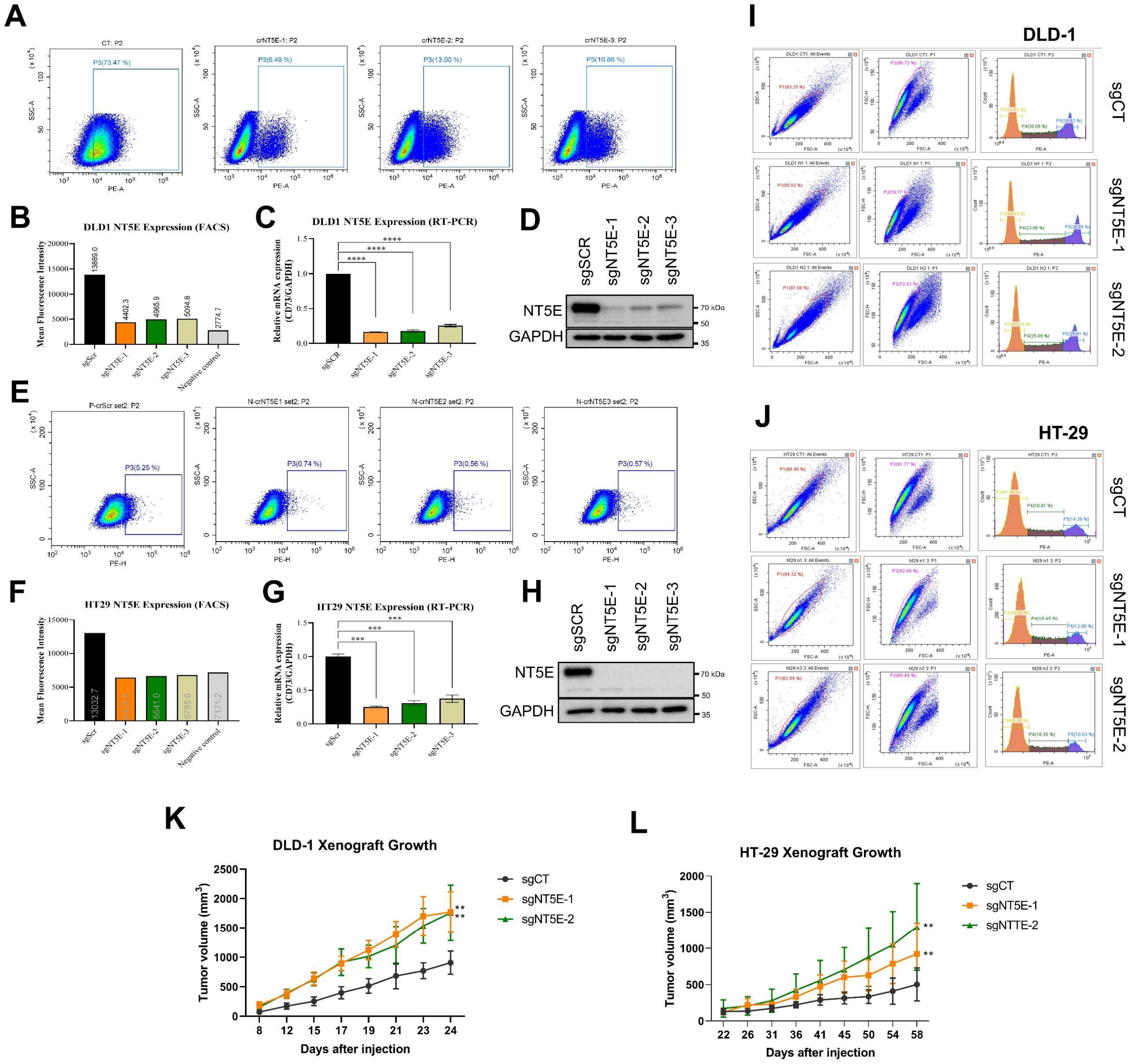

Supplementary Figure 2. Supplementary information for Figure 3. CRISPR/Cas9-mediated (sgRNA) NT5E/CD73 depletion in DLD-1 cells via **(A** and **B)** flow cytometry, **(C)** RT-PCR and **(D)** WB. CRISPR/Cas9-mediated (sgRNA) NT5E/CD73 depletion in HT-29 cells via **(E** and **F)** flow cytometry, **(G)** RT-PCR and **(H)** WB. Cell cycle analysis in **(I)** DLD-1 and **(J)** HT-29 cells upon NT5E/CD73 depletion via sgRNA. **(K&L)** Xenograft tumor growth graph related to tumors shown in Figure 3K and 3L.

Figure S3

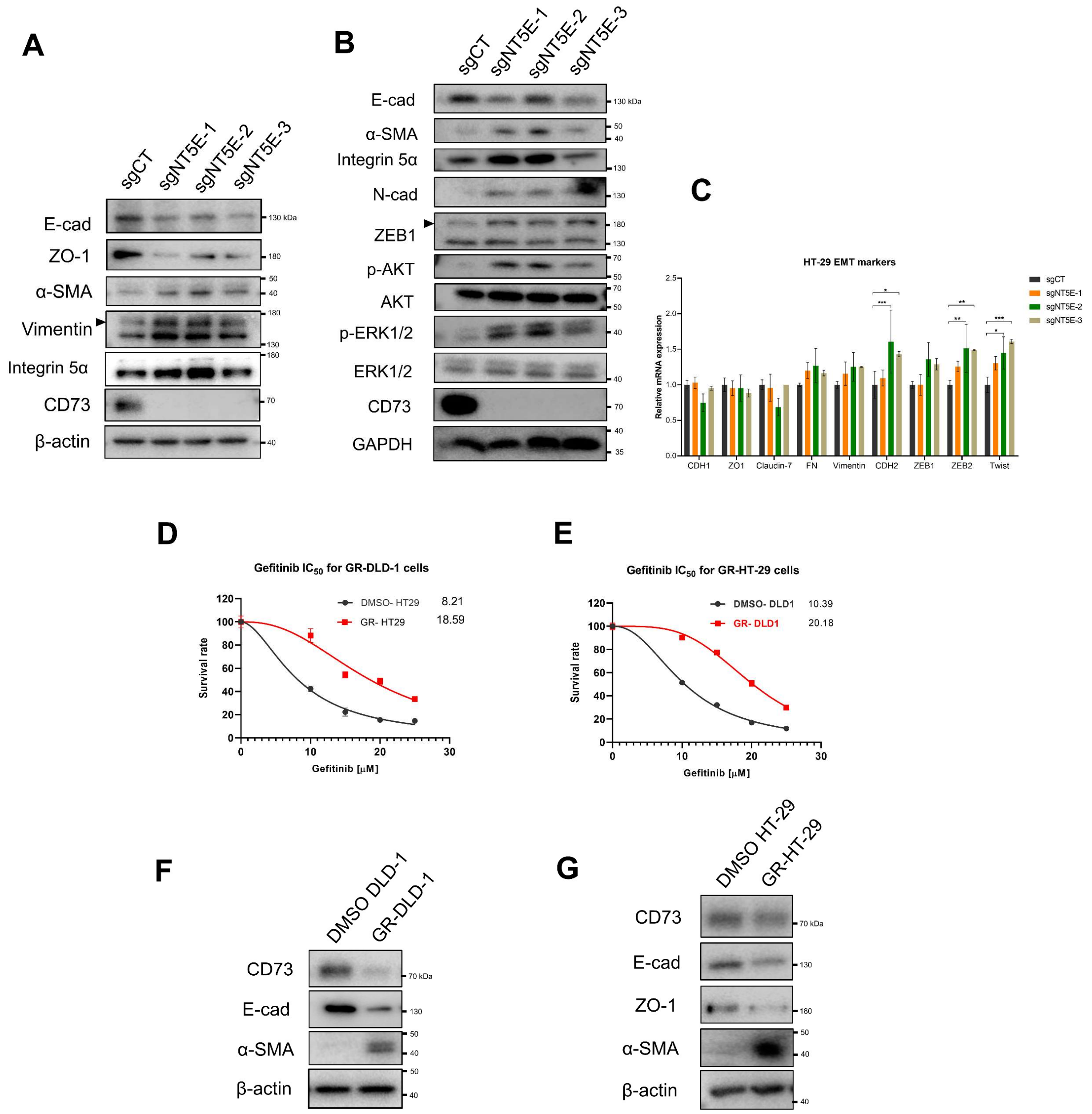

Supplementary Figure 3. Supplementary information for Figure 4. Expression profiles for some EMT marker levels in CD73 deficient **(A)** DLD-1 and **(B)** HT-29 cells via WB. **(C)** EMT marker expression in CD73-deficient HT-29 cells were also assessed via RT-PCR. (**D and E**) Calculation of gefitinib IC_{50} values in gefitinib resistant (GR) CRC cell lines.

All data were presented from left to right for DLD1, HT-29, SW480 respectively. **(F and G)** Expression of some EMT markers in control vs GR CRC cells.

Figure S4

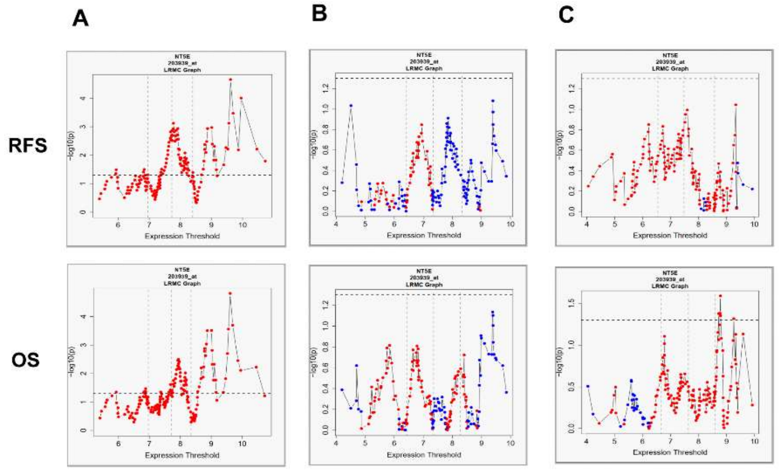

D

		p	HR	95% CI for HR Lower	Upper
**Step 1**	Stromal score (Estimate)*	0.004	1.354	1.103	1.661
	NT5E (203939_at)**	0.013	1.607	1.104	2.338

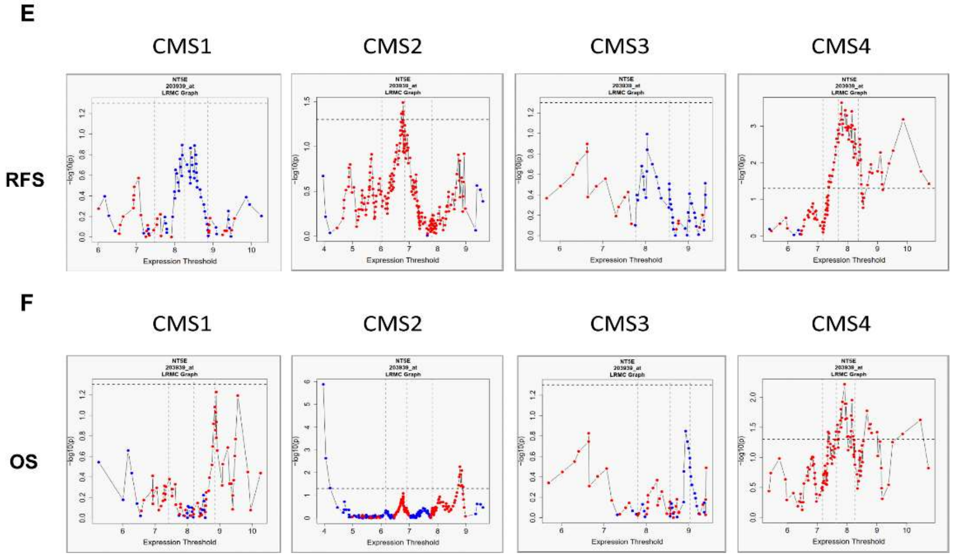

Supplementary Figure 4. Supplementary information for Figure 7. CD73/NT5E high expression is associated with poor recurrence-free survival and overall survival in samples only with **(A)** high stroma score (Stroma-high) yet no such association is observed with **(B)** intermediate and **(C)** low stroma scores according to LRMC graphs. (D) **Multivariate cox regression analyses with stromal score (RFS).** * Samples were sorted based on Estimate's stromal score. 188, 189 and 189 samples were categorized as stromal score low (assigned to 1), intermediate (assigned to 2) and high (assigned to 3), respectively. Assigned values for these categories (1-2-3) were used as continuous variables in MVA. Patients with available non-zero survival time were included in the analysis. (HR: hazard ratio). ** The threshold within interquartile range that gives the lowest log-rank p value (6.96) was used to categorize low and high expression groups. **(E)** RFS and **(F)** OS were used as clinical outcome. Log-rank multiple cut-off graph shows gene expression-based cut-offs on x axis and log-rank p values at each specific cut-off on y axis (see Methods). Blue and red colors indicate association with good and poor prognosis, respectively. Vertical dashed lines represent 25 percentile, median and 75th percentile values. Horizontal dashed line indicates 0.05 p value.

Figure S5

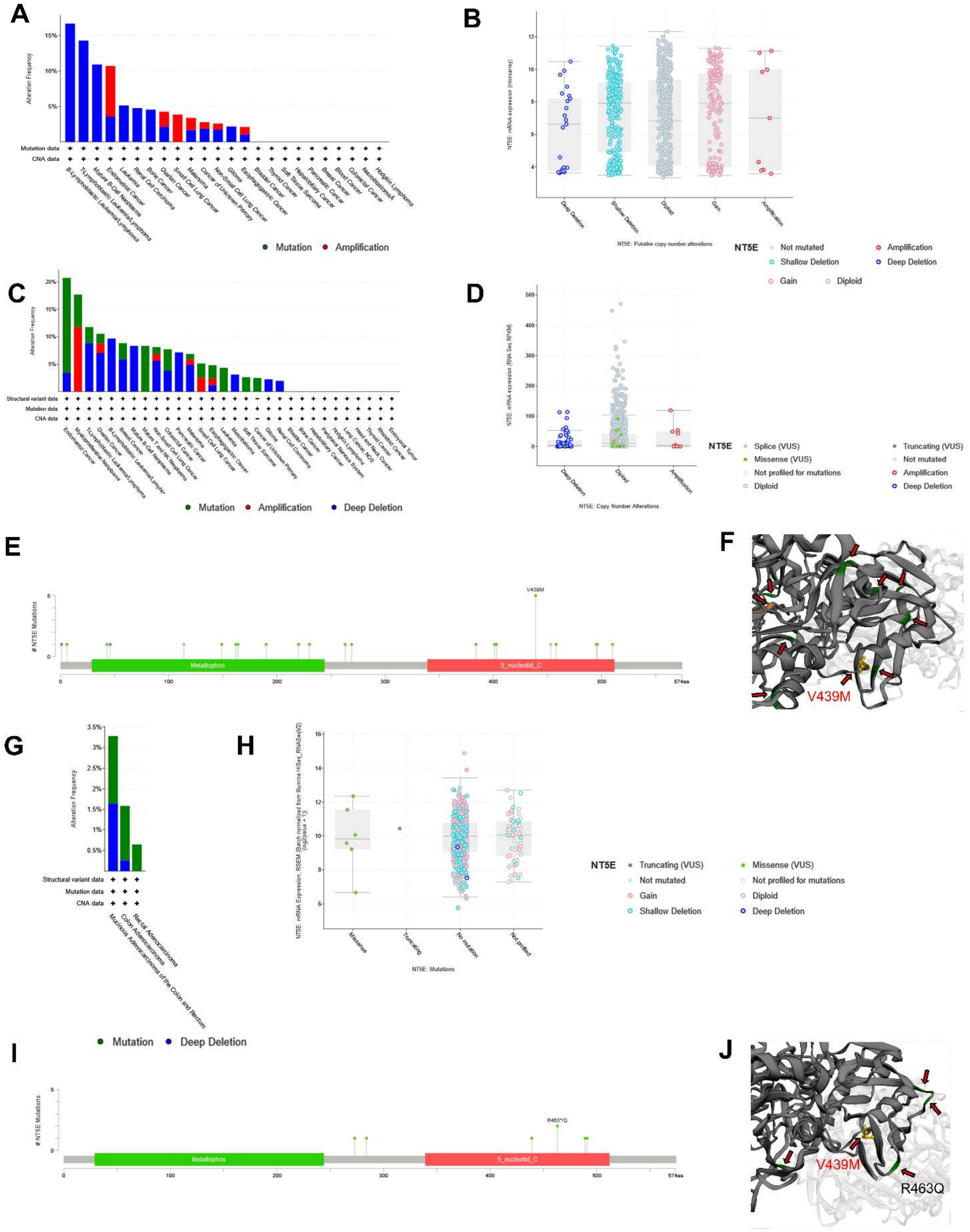
A
B
C
D
E
F
G
H
I
J
Mutation
Amplification
Deep Deletion
NT5E
V439M
R463Q
Metallophos
5_nucleotid_C
Alteration Frequency
NT5E Mutations
NT5E: Putative copy-number alterations
NT5E: Copy Number Alterations
NT5E: Mutations
Not mutated
Shallow Deletion
Gain
Diploid
Splice (VUS)
Missense (VUS)
Not profiled for mutations
Truncating (VUS)
Structural variant data
Mutation data
CNA data
Rectal Adenocarcinoma
Colon Adenocarcinoma
Mucinous Adenocarcinoma of the Colon and Rectum

Supplementary Figure 5. Supplementary information for Figure 7. Mutational alterations in NT5E were enumerated in cBioPortal by the use of datasets from **(A)** TCGA and **(C)** CCLE datasets (all cancers). Similarly, **(B)** TCGA and **(D)** CCLE datasets were used for analyzing NT5E copy number alterations according to RNA-Seq data in all cancers. **(E)** Frequently observed NT5E mutations in all cancers patients from TCGA sets were illustrated as output from cBioPortal. **(F)** Some of the most frequently observed mutations accumulate around CD73 catalytic cleft (most abundant mutation V439M is marked in red). **(G)** Mutations and **(H)** copy number alterations in TCGA colorectal cancer datasets were visualized via cBioPortal. **(I)** Frequently observed NT5E mutations in CRC patients from TCGA sets were illustrated as output from cBioPortal. **(J)** Similar to results from all cancers, some of the most frequently observed mutations accumulate around CD73 catalytic cleft in tumors from CRC patients (V439M is again most frequent mutation in CRC patients). All the data and analysis in this figure were carried and visualized by using cBioPortal.

Figure S6

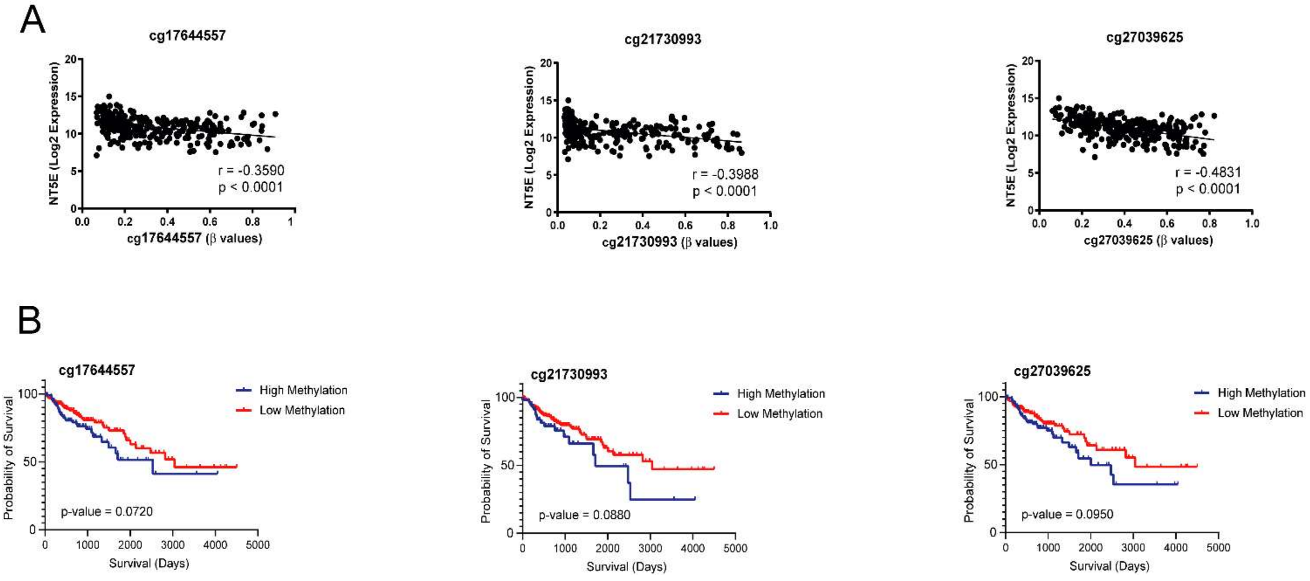

Supplementary Figure 6. Supplementary information for Figure 7. NT5E promotor methylation is correlated with lower NT5E expression and worse prognosis in TCGA COAD patients. Additional methylation specific analysis for NT5E promoter sites. **(A)** Scatter plot showing NT5E expression and methylation β levels for cg17644557, cg21730993 and cg27039625 CpG. Pearson correlation r and p values are given. **(B)** KM-plot of patients with higher methylation of NT5E promotor at cg17644557, cg21730993, and cg27039625 had relatively worse survival, yet none of these prognostic associations are significant (p values are 0.072; 0.088 or 0.095 respectively).

3 Supplementary Tables

Supplementary Table 1: Multivariate cox regression (MVA) analyses for determination of confounding factors for the effect of CD73 expression on RFS of CRC patients (formatted results). Samples were sorted based on indicated factors in each group (see supplementary table 2 for detailed analysis). Patients with available non-zero survival time were included in the analysis. As a result of this MVA, High NT5E expression was found to be predictor of worse prognosis independent of treatment, age, KRAS or BRAF mutation status and tumor location while it was still dependent on MSI (MMR status) and TNM Stage. HR: Hazard ratio, Sig.: p value of cox regression (significant p values are marked with red).* Adjuvant chemotherapy; ** TNM Stage: Treated as a continuous variable (1, 2, 3, 4); *** Samples are considered as wild type if only both genes are wild type, other situations are considered mutated; ****The threshold within interquartile range that gives the lowest log-rank p value (6.96) was used to categorize low and high expression groups.

				95,0% CI for Exp(B)	
	n	Sig. (p)	HR	Lower	Upper
Treatment*		,316	,810	,537	1,222
Not received:	298				
Received	220				
Age		,812	,951	,625	1,445
equal or above 60	383				
below 60	135				
MMR Status		**,021**	,432	,212	,879
p MMR	405				
d MMR	71				
NA	43				
TNM Stage**		**,000**	1,694	1,286	2,232
0	4				
1	32				
2	253				
3	200				
4	30				
Tumor location		,119	1,384	,919	2,085
proximal	209				
distal	310				
KRAS or BRAF mutation ***		,148	1,350	,899	2,028
Mutant	241				
Wild type	235				
NT5E (203939_at)****		**,027**	1,645	1,057	2,561
High	342				
Low	177				

Supplementary Table 2: Related to Supplementary Table 1. Multivariate cox regression (MVA) analyses with confounding factors for RFS in CRC (detailed results of the analysis presented in Supplementary Table 1).

Variables in the Equation

		B	SE	Wald	df	Sig.	Exp(B)	95,0% CI for Exp(B)	
								Lower	Upper
Step 1	TREATMENT	-,210	,210	1,005	1	,316	,810	,537	1,222
	AGE60	-,051	,214	,056	1	,812	,951	,625	1,445
	MSI	-,840	,363	5,363	1	,021	,432	,212	,879
	TNM@STAGE	,527	,141	14,043	1	,000	1,694	1,286	2,232
	tumor.location	,325	,209	2,425	1	,119	1,384	,919	2,085
	krasORbrafMUT	,300	,208	2,094	1	,148	1,350	,899	2,028
	NT5E203939bestLRMCrfs	,498	,226	4,861	1	,027	1,645	1,057	2,561
Step 2	TREATMENT	-,199	,204	,948	1	,330	,820	,549	1,223
	MSI	-,836	,362	5,320	1	,021	,433	,213	,882
	TNM@STAGE	,528	,141	14,046	1	,000	1,695	1,286	2,234
	tumor.location	,328	,208	2,478	1	,115	1,388	,923	2,089
	krasORbrafMUT	,298	,207	2,066	1	,151	1,347	,897	2,022
	NT5E203939bestLRMCrfs	,499	,226	4,880	1	,027	1,647	1,058	2,565
Step 3	MSI	-,790	,360	4,831	1	,028	,454	,224	,918
	TNM@STAGE	,472	,131	12,987	1	,000	1,603	1,240	2,072
	tumor.location	,310	,207	2,239	1	,135	1,364	,908	2,048
	krasORbrafMUT	,303	,206	2,160	1	,142	1,354	,904	2,029
	NT5E203939bestLRMCrfs	,498	,225	4,885	1	,027	1,645	1,058	2,557
Step 4	MSI	-,765	,359	4,537	1	,033	,465	,230	,941
	TNM@STAGE	,490	,131	13,935	1	,000	1,632	1,262	2,111
	tumor.location	,219	,199	1,211	1	,271	1,244	,843	1,836
	NT5E203939bestLRMCrfs	,581	,218	7,140	1	,008	1,788	1,168	2,739
Step 5	MSI	-,857	,349	6,031	1	,014	,424	,214	,841
	TNM@STAGE	,481	,131	13,394	1	,000	1,617	1,250	2,092
	NT5E203939bestLRMCrfs	,549	,216	6,470	1	,011	1,731	1,134	2,642

Supplementary Table 3: Linear correlation of CD73/NT5E with EMT markers in dataset GSE39582 according to immune infiltration status (high, intermediate or low). Pearson correlation coefficients are presented in the table, positive correlations are denoted by shades of red and negative correlations are denoted in shades of green, according to Microsoft Excel's heatmap function (significant correlations ($p<0.05$) were marked by bold numbers, for p values please see supplementary table 3). CDH1, an epithelial marker, was included for comparison.

**IMMUNE SCORE**	**EMT**	**MARKERS**	NT5E 203939_at	NT5E 227486_at	NT5E 1553994_at	NT5E 1553995_a_at
HIGH	SNAI1	219480_at	-0,030	**0.183**	0.021	0.047
	SNAI2	213139_at	**0.231**	**0.277**	**0.151**	**0.242**
	TWIST1	213943_at	0.127	**0.320**	0.092	0.087
	TWIST2	229404_at	0.015	**0.294**	0.116	0.091
	VIM	201426_s_at	**0.186**	**0.376**	0.023	**0.152**
	ZEB1	212764_at	**0.275**	**0.388**	0.109	0.092
	ZEB2	203603_s_at	**0.228**	**0.351**	**0.203**	**0.178**
	CDH1	201130_s_at	**-0.224**	**-0.406**	**-0.200**	-0.071
INT	SNAI1	219480_at	**-0.272**	0.007	-0.262	-0.235
	SNAI2	213139_at	0.134	**0.165**	0.088	0.097
	TWIST1	213943_at	-0.012	**0.261**	-0.016	-0.080
	TWIST2	229404_at	0.033	**0.317**	0.104	0.027
	VIM	201426_s_at	-0.020	**0.197**	**-0.151**	-0.090
	ZEB1	212764_at	**0.175**	**0.309**	-0.015	-0.024
	ZEB2	203603_s_at	0.099	**0.259**	0.044	0.020
	CDH1	201130_s_at	-0.026	-0.136	0.022	0.123
LOW	SNAI1	219480_at	**-0.143**	-0.115	-0.090	-0.079
	SNAI2	213139_at	-0.085	-0.094	0.049	0.009
	TWIST1	213943_at	-0.094	-0.029	-0.020	-0.057
	TWIST2	229404_at	-0.071	-0.007	-0.023	-0.068
	VIM	201426_s_at	-0.131	-0.101	-0.056	-0.046
	ZEB1	212764_at	-0.140	-0.056	-0.050	-0.093
	ZEB2	203603_s_at	0.035	0.024	0.101	0.019
	CDH1	201130_s_at	**0.156**	0.008	**0.206**	**0.274**

Supplementary Table 4: Linear correlation of CD73/NT5E with EMT markers in dataset GSE39582 according to stroma density status (high, intermediate or low). Pearson correlation coefficients are presented in the table, positive correlations are denoted by shades of red and negative correlations are denoted in shades of green, according to Microsoft Excel's heatmap function (significant correlations ($p<0.05$) were marked by bold numbers, for p values please see supplementary table 4).

STROMA SCORE	EMT MARKERS		NT5E 203939_at	NT5E 227486_at	NT5E 1553994_at	NT5E 1553995_a_at
HIGH	SNAI1	219480_at	0.023	**0.144**	0.107	0.098
	SNAI2	213139_at	**0.282**	**0.220**	**0.251**	**0.304**
	TWIST1	213943_at	0.093	**0.284**	**0.154**	0.053
	TWIST2	229404_at	0.045	**0.315**	**0.231**	0.141
	VIM	201426_s_at	**0.279**	**0.398**	**0.155**	**0.247**
	ZEB1	212764_at	**0.257**	**0.398**	0.116	0.029
	ZEB2	203603_s_at	**0.305**	**0.335**	**0.329**	**0.258**
	CDH1	201130_s_at	**-0.177**	**-0.413**	-0.122	0.013
INT	SNAI1	219480_at	**-0.195**	-0.035	-0.135	**-0.160**
	SNAI2	213139_at	**0.265**	0.116	**0.183**	**0.199**
	TWIST1	213943_at	0.100	0.088	0.125	0.053
	TWIST2	229404_at	0.016	0.058	0.074	-0.012
	VIM	201426_s_at	0.095	0.050	-0.048	0.042
	ZEB1	212764_at	**0.151**	0,071	0.022	-0.025
	ZEB2	203603_s_at	**0.190**	**0.183**	0.126	0.071
	CDH1	201130_s_at	0.028	-0.043	0.033	0.141
LOW	SNAI1	219480_at	**-0.283**	**-0.190**	**-0.251**	**-0.193**
	SNAI2	213139_at	-0.040	-0.104	**0.178**	0.132
	TWIST1	213943_at	-0.099	-0.024	-0.020	-0.048
	TWIST2	229404_at	-0.025	0.013	0.096	0.051
	VIM	201426_s_at	-0.041	-0.066	0.063	0.097
	ZEB1	212764_at	0.030	-0.004	0.073	0.069
	ZEB2	203603_s_at	0.117	0.054	**0.265**	**0.198**
	CDH1	201130_s_at	0.021	-0.089	0.063	0.129

Supplementary Table 5: Related to Supplementary Table 3. Pearson p values for linear correlation of CD73/NT5E with EMT markers in dataset GSE39582 according to immune infiltration status (high, intermediate or low). p values below 0.05 are shown in red.

IMMUNE SCORE	EMT MARKERS		NT5E 203939_at	NT5E 227486_at	NT5E 1553994_at	NT5E 1553995_a_at
HIGH	SNAI1	219480_at	0.683	0.012	0.773	0.520
	SNAI2	213139_at	0.001	<0.001	0.038	0.001
	TWIST1	213943_at	0.081	<0.001	0.207	0.233
	TWIST2	229404_at	0.835	<0.001	0.112	0.212
	VIM	201426_s_at	0.010	<0.001	0.751	0.037
	ZEB1	212764_at	<0.001	<0.001	0.137	0.210
	ZEB2	203603_s_at	0.002	<0.001	0.005	0.014
	CDH1	201130_s_at	0.002	<0.001	0.006	0.330
INT	SNAI1	219480_at	<0.001	0.922	<0.001	0.001
	SNAI2	213139_at	0.066	0.023	0.226	0.183
	TWIST1	213943_at	0.868	<0.001	0.831	0.276
	TWIST2	229404_at	0.651	<0.001	0.154	0.708
	VIM	201426_s_at	0.786	0.007	0.038	0.218
	ZEB1	212764_at	0.016	<0.001	0.840	0.746
	ZEB2	203603_s_at	0.174	<0.001	0.544	0.781
	CDH1	201130_s_at	0.718	0.062	0.764	0.093
LOW	SNAI1	219480_at	0.050	0.115	0.218	0.281
	SNAI2	213139_at	0.248	0.201	0.503	0.897
	TWIST1	213943_at	0.198	0.693	0.786	0.435
	TWIST2	229404_at	0.335	0.919	0.759	0.352
	VIM	201426_s_at	0.072	0.166	0.445	0.530
	ZEB1	212764_at	0.056	0.445	0.498	0.205
	ZEB2	203603_s_at	0.632	0.745	0.170	0.797
	CDH1	201130_s_at	0.032	0.915	0.005	<0.001

Supplementary Table 6: Related to Supplementary Table 4. Pearson p values for linear correlation of CD73/NT5E with EMT markers in dataset GSE39582 according to stroma density status (high, intermediate or low). p values below 0.05 are shown in red.

STROMA SCORE	EMT MARKERS		NT5E 203939_at	NT5E 227486_at	NT5E 1553994_at	NT5E 1553995_a_at
HIGH	SNAI1	219480_at	0.752	0.048	0.145	0.180
	SNAI2	213139_at	<0.001	0.002	<0.001	<0.001
	TWIST1	213943_at	0.204	<0.001	0.034	0.470
	TWIST2	229404_at	0.541	<0.001	0.001	0.053
	VIM	201426_s_at	<0.001	<0.001	0.033	0.001
	ZEB1	212764_at	<0.001	<0.001	0.112	0.689
	ZEB2	203603_s_at	<0.001	<0.001	<0.001	<0.001
	CDH1	201130_s_at	0.015	<0.001	0.093	0.861
INT	SNAI1	219480_at	0.007	0.631	0.064	0.028
	SNAI2	213139_at	<0.001	0.112	0.012	0.006
	TWIST1	213943_at	0.171	0.226	0.087	0.468
	TWIST2	229404_at	0.829	0.430	0.309	0.875
	VIM	201426_s_at	0.193	0.494	0.512	0.564
	ZEB1	212764_at	0.038	0.334	0.762	0.729
	ZEB2	203603_s_at	0.009	0.012	0.085	0.330
	CDH1	201130_s_at	0.700	0.556	0.654	0.054
LOW	SNAI1	219480_at	<0.001	0.009	0.001	0.008
	SNAI2	213139_at	0.587	0.156	0.014	0.070
	TWIST1	213943_at	0.175	0.746	0.789	0.515
	TWIST2	229404_at	0.738	0.862	0.190	0.489
	VIM	201426_s_at	0.580	0.371	0.393	0.187
	ZEB1	212764_at	0.688	0.959	0.317	0.348
	ZEB2	203603_s_at	0.109	0.459	<0.001	0.006
	CDH1	201130_s_at	0.780	0.223	0.387	0.077

4 References

Calon A, Espinet E, Palomo-Ponce S, Tauriello DV, Iglesias M, Céspedes MV, Sevillano M, Nadal C, Jung P, Zhang XH, Byrom D, Riera A, Rossell D, Mangues R, Massagué J, Sancho E, Batlle E. (2012) Dependency of colorectal cancer on a TGF-β-driven program in stromal cells for metastasis initiation. *Cancer Cell* **22**(5):571-84. doi: 10.1016/j.ccr.2012.08.013

Chandrashekar,DS, Karthikeyan SK, Korla PK, Patel H, Shovon AR, Athar M, Netto GJ, Qin ZS, Kumar S, Manne U, Creighton CJ, Varambally S. (2022) UALCAN: An update to the integrated cancer data analysis platform. *Neoplasia (New York, N.Y.)* **25**, 18–27 10.1016/j.neo.2022.01.001

Che LH, Liu JW, Huo JP, Luo R, Xu RM, He C, Li YQ, Zhou AJ, Huang P, Chen YY, Ni W, Zhou YX, Liu YY, Li HY, Zhou R, Mo H, Li JM (2021) A single-cell atlas of liver metastases of colorectal cancer reveals reprogramming of the tumor microenvironment in response to preoperative chemotherapy. *Cell Discovery* **7**;7(1):80. doi: 10.1038/s41421-021-00312-y

Chen B, Khodadoust MS, Liu CL, Newman AM, Alizadeh AA (2018). Profiling Tumor Infiltrating Immune Cells with CIBERSORT. *Methods in Molecular Biology* **1711**:243-259. doi: 10.1007/978-1-4939-7493-1_12.

Colaprico A, Silva TC, Olsen C, Garofano L, Cava C, Garolini D, Sabedot TS, Malta TM, Pagnotta SM, Castiglioni I, Ceccarelli M, Bontempi G, Noushmehr H (2016) TCGAbiolinks: an R/Bioconductor package for integrative analysis of TCGA data. *Nucleic Acids Research* **44**(8):e71. doi: 10.1093/nar/gkv1507

Demirkol S, Gomceli I, Isbilen M, Dayanc BE, Tez M, Bostanci EB, Turhan N, Akoglu M, Ozyerli E, Durdu S, Konu O, Nissan A, Gonen M, Gure AO (2017) A Combined ULBP2 and SEMA5A Expression Signature as a Prognostic and Predictive Biomarker for Colon Cancer. *Journal of Cancer* **8**: 1113–1122, doi:10.7150/jca.17872

Gao J, Aksoy BA, Dogrusoz U, Dresdner G, Gross B, Sumer SO, Sun Y, Jacobsen A, Sinha R, Larsson E, Cerami E, Sander C, Schultz N (2013) Integrative analysis of complex cancer genomics and clinical profiles using the cBioPortal. Sci Signal **6**(269):pl1. doi: 10.1126/scisignal.2004088

Gautier L, Cope L, Bolstad BM, Irizarry RA (2004) affy--analysis of Affymetrix GeneChip data at the probe level. *Bioinformatics (Oxford, England)* **20**: 307–315, doi:10.1093/bioinformatics/btg405

Hao Y, Hao S, Andersen-Nissen E, Mauck WM, Zheng S, Butler A, Lee MJ, Wilk AJ, Darby C, Zager M, Hoffman P, Stoeckius M, Papalexi E, Mimitou EP, Jain J, Srivastava A, Stuart T, Fleming LM, Yeung B, Rogers AJ, McElrath JM, Blish CA, Gottardo R, Smibert P, Satija R (2021) Integrated analysis of multimodal single-cell data. *Cell* **184**: 3573-3587.e29, doi:10.1016/j.cell.2021.04.048

Haun RS, Quick CM, Siegel ER, Raju I, Mackintosh SG, Tackett AJ. Bioorthogonal labeling cell-surface proteins expressed in pancreatic cancer cells to identify potential diagnostic/therapeutic biomarkers (2015). *Cancer Biology & Therapy* **16**(10):1557-65. doi: 10.1080/15384047.2015.1071740.

The Human Protein Atlas Colorectal Cancer Protein Expression: Antibody Staining for Antibody HPA017357. https://www.proteinatlas.org/ENSG00000135318-NT5E/cancer/colorectal+cancer#img

Love MI, Huber W, Anders S (2014) Moderated estimation of fold change and dispersion for RNA-seq data with DESeq2. *Genome biology* **15**: 550, doi:10.1186/s13059-014-0550-8

Marisa L, Reyniès A de, Duval A, Selves J, Gaub MP, Vescovo L, Etienne-Grimaldi M-C, Schiappa R, Guenot D, Ayadi M, Kirzin S, Chazal M, Fléjou J-F, Benchimol D, Berger A, Lagarde A, Pencreach E, Piard F, Elias D, Parc Y, Olschwang S, Milano G, Laurent-Puig P, Boige V (2013) Gene expression classification of colon cancer into molecular subtypes: characterization, validation, and prognostic value. *PLoS medicine* **10**: e1001453, doi:10.1371/journal.pmed.1001453

McGinnis CS, Murrow LM, Gartner ZJ (2019) DoubletFinder: Doublet Detection in Single-Cell RNA Sequencing Data Using Artificial Nearest Neighbors. *Cell Systems* **8**(4):329-337.e4. doi: 10.1016/j.cels.2019.03.003

Newman AM, Liu CL, Green MR, Gentles AJ, Feng W, Xu Y, Hoang CD, Diehn M, Alizadeh AA (2015). Robust enumeration of cell subsets from tissue expression profiles. *Nature Methods* **12**(5):453-7. doi: 10.1038/nmeth.3337.

Pelka K, Hofree M, Chen JH, Sarkizova S, Pirl JD, Jorgji V, Bejnood A, Dionne D, Ge WH, Xu KH, Chao SX, Zollinger DR, Lieb DJ, Reeves JW, Fuhrman CA, Hoang ML, Delorey T, Nguyen LT, Waldman J, Klapholz M, Wakiro I, Cohen O, Albers J, Smillie CS, Cuoco MS, Wu J, Su MJ, Yeung J, Vijaykumar B, Magnuson AM, Asinovski N, Moll T, Goder-Reiser MN, Applebaum AS, Brais LK, DelloStritto LK, Denning SL, Phillips ST, Hill EK, Meehan JK, Frederick DT, Sharova T, Kanodia A, Todres EZ, Jané-Valbuena J, Biton M, Izar B, Lambden CD, Clancy TE, Bleday R, Melnitchouk N, Irani J, Kunitake H, Berger DL, Srivastava A, Hornick JL, Ogino S, Rotem A, Vigneau S, Johnson BE, Corcoran RB, Sharpe AH, Kuchroo VK, Ng K, Giannakis M, Nieman LT, Boland GM, Aguirre AJ, Anderson AC, Rozenblatt-Rosen O, Regev A, Hacohen N (2021) Spatially organized multicellular immune hubs in human colorectal cancer. Cell **184**(18):4734-4752.e20. doi: 10.1016/j.cell.2021.08.003

Smith JJ, Deane NG, Wu F, Merchant NB, Zhang B, Jiang A, Lu P, Johnson JC, Schmidt C, Bailey CE, Eschrich S, Kis C, Levy S, Washington MK, Heslin MJ, Coffey RJ, Yeatman TJ, Shyr Y, Beauchamp RD (2010) Experimentally derived metastasis gene expression profile predicts recurrence and death in patients with colon cancer. *Gastroenterology* **138**: 958–968, doi:10.1053/j.gastro.2009.11.005

Yoshihara K, Shahmoradgoli M, Martínez E, Vegesna R, Kim H, Torres-Garcia W, Treviño V, Shen H, Laird PW, Levine DA, Carter SL, Getz G, Stemke-Hale K, Mills GB, Verhaak RGW (2013) Inferring tumour purity and stromal and immune cell admixture from expression data. *Nature communications* **4**: 2612, doi:10.1038/ncomms3612
